# Emerging 2D Ferroelectric Semiconductors: From Fundamentals to Advanced Device Applications

**DOI:** 10.1002/advs.202514185

**Published:** 2025-11-03

**Authors:** Mengshuang Chi, Xiang Zhang, JiTao Liu, YiFan Wang, Aifang Yu, Di Guo, Junyi Zhai

**Affiliations:** ^1^ Beijing Key Laboratory of Micro‐Nano Energy and Sensor Center for High‐Entropy Energy and Systems Beijing Institute of Nanoenergy and Nanosystems Chinese Academy of Sciences Beijing 101400 P. R. China; ^2^ School of Nanoscience and Engineering University of Chinese Academy of Sciences Beijing 100049 P. R. China; ^3^ Beijing Huairou Laboratory Beijing 100049 P. R. China

**Keywords:** 2D materials, electronics, ferroelectric semiconductors, optoelectronics, polarization mechanisms

## Abstract

Two‐dimensional (2D) ferroelectric semiconductors, as an emerging class of functional materials, attract considerable interest in nanoelectronics, spintronics, and optoelectronics, owing to their unique ability to combine ferroelectricity and semiconducting properties at the ultimate thickness limit. This review provides a comprehensive overview of the development, fundamental mechanisms, and recent advances of 2D ferroelectric semiconductors. The origins and unique characteristics of 2D ferroelectricity are discussed, and representative intrinsic 2D ferroelectric semiconductors as well as extrinsic systems are summarized. The potential applications of these materials in electronics, optoelectronics, spintronics, and valleytronics are discussed in detail. Finally, the key challenges facing the field are outlined, and perspectives on future directions are offered. This review aims to provide a systematic reference for both fundamental studies and technological development, fostering the advancement of 2D ferroelectric semiconductors toward high‐performance and multifunctional device applications.

## Graphical abstract

This review highlights the emerging field of two‐dimensional ferroelectric semiconductors, focusing on their quantum‐scale polarization mechanisms, material innovations, and diverse device applications. It also summarizes key challenges and outlines future directions, including strain and twist‐angle engineering, quantum coupling, and flexible electronics for next‐generation low‐power nanoelectronic systems.

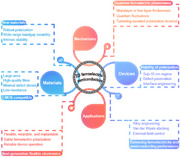

## 1 Introduction

Ferroelectricity, defined as the presence of a spontaneous and reversible electric polarization, was first discovered in 1920 by Joseph Valasek in Rochelle salt (potassium sodium tartrate).^[^ ^1^ ^]^ Below a characteristic Curie temperature (*T_c_*), these materials exhibit a non‐zero polarization that can be reversed by applying an external electric field, leading to a hysteretic polarization–field loop. This reversible polarization makes ferroelectrics direct analogs of ferromagnets (hence the prefix “ferro‐”), with analogous features such as remnant polarization and coercive fields. After Valasek's pioneering work, other early ferroelectrics were identified: for example, KDP (KH_2_PO_4_) was found in the 1930s to become ferroelectric below ≈122 K.^[^ ^2^ ^]^ However, these compounds were characterized by hydrogen‐bonded crystal structures, high water solubility, and poor mechanical stability, limiting practical use. Consequently, ferroelectricity was initially considered a niche phenomenon, with limited utility and theoretical interest during its formative decades. The field took a major turn with the discovery of robust, room‐temperature ferroelectricity in inorganic oxides. In particular, BaTiO_3_ was found in the mid‐1940s to undergo a ferroelectric transition near 120 °C.^[^ ^3^ ^]^ This stable perovskite oxide, characterized by a very high dielectric constant, enabled the development of the first ferroelectric devices, including ultrasound transducers and, subsequently, non‐volatile memory systems. Subsequent work in the 1950s–1960s expanded this family: lead zirconate titanate (Pb(Zr, Ti)O_3_, or PZT) and related solid solutions became the workhorses for piezoelectric and ferroelectric applications. The simple perovskite structure of BaTiO_3_ and its derivatives also made them ideal for developing the phenomenological Landau–Devonshire theory of ferroelectricity (double‐well potential models), which remains a cornerstone for understanding ferroelectric phase transitions and polarization switching.

Throughout the late 20th century, ferroelectric research focused on improving material properties (higher *T_c_*, larger polarization, fatigue resistance) and device integration. Despite these advances, a fundamental limitation emerged: when ferroelectric films are thinned toward the nanometer scale, the depolarization field (arising from incomplete screening of surface charges) suppresses polarization. Theoretical studies predicted that below a critical thickness (often only a few unit cells), a conventional ferroelectric will lose its polar order.^[^ ^4^, ^5^ ^]^ Experimentally, ferroelectricity in perovskite thin films typically vanishes below a few nanometers unless elaborate interface engineering is used to compensate for the depolarizing field.^[^ ^6^, ^7^ ^]^ These scaling limits, together with the complex fabrication of oxide films, have driven the search for novel atomically thin ferroelectrics.

A breakthrough came with the discovery that layered two‐dimensional (2D) semiconductors can host intrinsic ferroelectricity. In contrast to oxide films, 2D layered materials have no dangling surface bonds, allowing clean, trap‐free interfaces and robust polar order down to a single layer. Early studies in the 1960s–1970s identified bulk ferroelectricity in IV–VI monochalcogenides^[^ ^8^ ^]^ (SnS, SnSe, GeS, and GeSe) and in transition‐metal phosphorus trisulfides^[^ ^9^ ^]^ (MPX_3_ compounds), but only recently have ultrathin 2D forms been explored. First‐principles calculations in 2016–2017 predicted that monolayers of certain van der Waals (vdW) materials (e.g., SnSe,^[^ ^10^ ^]^ GeTe,^[^ ^11^ ^]^ In_2_Se_3_^[^ ^12^ ^]^) possess spontaneous polarization arising from in‐plane (IP) ionic displacement or interlayer sliding. A key advance was achieved in 2016, when room‐temperature switchable spontaneous polarization was experimentally demonstrated in ultrathin 4 nm CuInP_2_S_6_ (CIPS),^[^ ^13^ ^]^ challenging the long‐held view that depolarization fields would suppress ferroelectricity at the nanoscale. Since then, a wide variety of 2D vdW ferroelectrics have been identified. These include NiI_2_,^[^ ^14^ ^]^ α‐In_2_Se_3_,^[^ ^15^ ^]^ CuCrP_2_S_6_,^[^ ^16^ ^]^ the family of group‐IV monochalcogenides^[^ ^17^, ^18^, ^19^ ^]^ (GeS, GeSe, SnS, SnSe, etc.), and layered transition‐metal dichalcogenides^[^ ^20^ ^]^ (e.g., MoTe_2_, WTe_2_). Very recently, layered bismuth oxychalcogenides^[^ ^21^ ^]^ (e.g., Bi_2_O_2_Se) and niobium oxyhalides^[^ ^22^ ^]^ (e.g., NbOI_2_) have been proposed as 2D ferroelectric semiconductors, where subtle lattice distortions break inversion symmetry. In addition to these intrinsic materials, researchers have used strain, charge doping, surface functionalization, and engineered defects to induce ferroelectricity in nominally non‐ferroelectric 2D crystals. The field is also moving toward “multiphysics” ferroelectrics: for example, heterostructures like In_2_Se_3_/CrI_3_^[^ ^23^ ^]^ couple ferroelectric and magnetic or valley degrees of freedom, opening new device possibilities.

A key advantage of many 2D ferroelectrics is that they are semiconductors with moderate band gaps. This means a single 2D crystal can simultaneously host a switchable polarization and support electronic conduction. In practice, 2D ferroelectric semiconductors overcome the scaling limits of conventional ferroelectrics: for example, α‐In_2_Se_3_ maintains robust polarization down to a few atomic layers. Moreover, the clean vdW surfaces allow easy stacking and gating without the dielectric fatigue problems of oxide devices. These properties enable novel device concepts. First, a 2D ferroelectric can serve simultaneously as an active transistor channel and a nonvolatile memory element. For example, α‐In_2_Se_3_ has been used as the channel in field‐effect transistors whose conductance state is set by the ferroelectric polarization. Because the channel polarization does not rely on a separate gate dielectric, these devices intrinsically combine logic and memory functions, with the transistor state being nonvolatile. Such devices have been demonstrated to operate with fast switching (tens of nanoseconds) and tunable neural‐network dynamics, pointing toward in‐memory and neuromorphic computing applications.^[^ ^24^ ^]^ Second, nonvolatile gating is now possible: a ferroelectric layer in a 2D heterostructure can gate an adjacent semiconductor layer without continuous power, enabling ultra‐low‐energy memory and logic. Third, the polar order in a semiconductor enables optoelectronic enhancements. For instance, broken inversion symmetry gives a bulk photovoltaic effect, where light absorption can generate photovoltages above the bandgap. It was recently shown that 2D CIPS exhibits a bulk photovoltaic response two orders of magnitude larger than classic oxide ferroelectrics.^[^ ^25^ ^]^ Similarly, α‐In_2_Se_3_ exhibits a strong pyroelectric response in conjunction with its intrinsic polarization, giving rise to a pyro‐photovoltaic effect that enables it to function simultaneously as a self‐powered photodetector and a memory element.^[^ ^26^ ^]^

**Figure 1:**
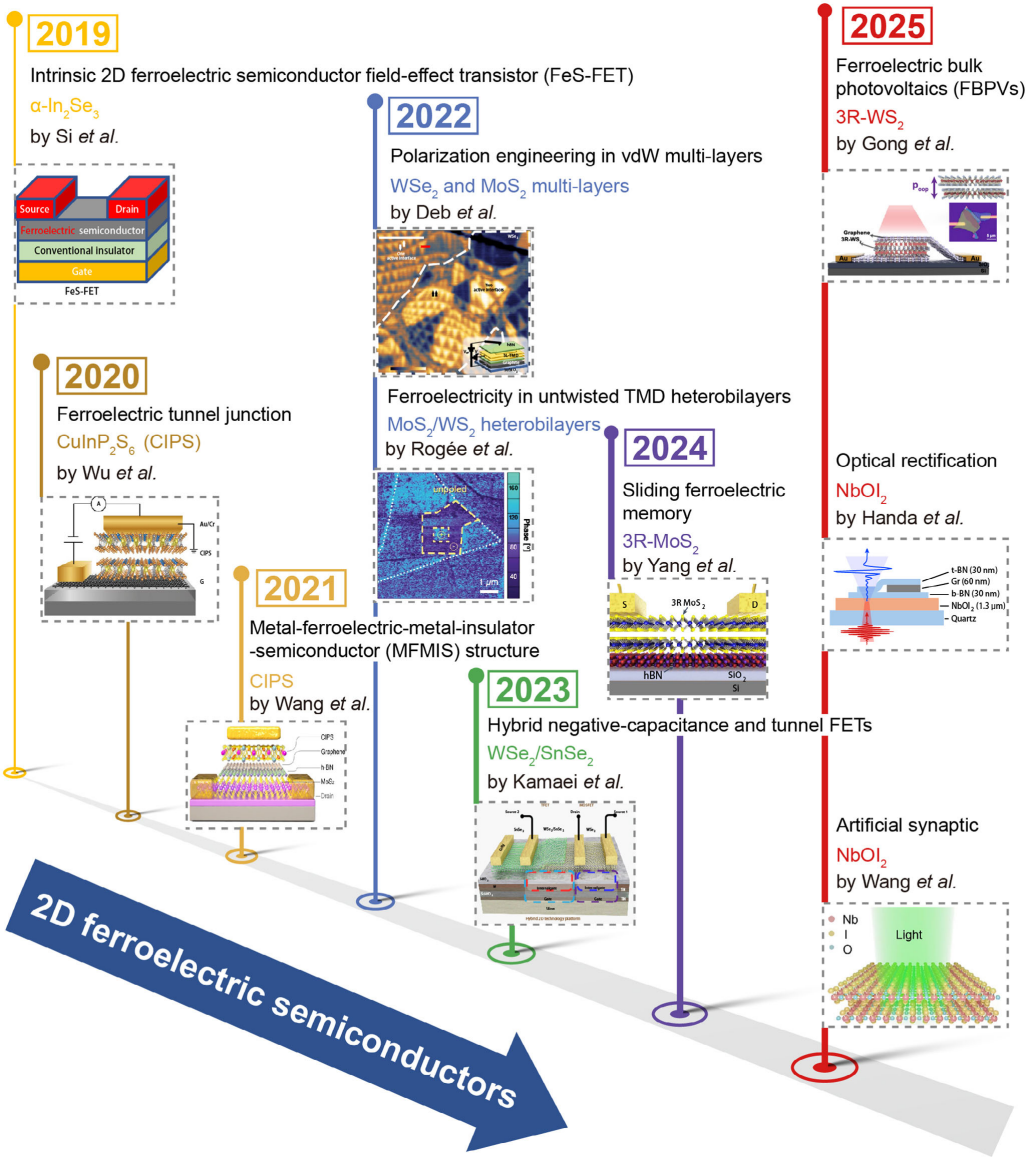
Timeline of milestones in 2D ferroelectric semiconductors. Reproduced with permission.^[^ ^27^, ^28^, ^29^, ^30^, ^31^, ^32^, ^33^, ^34^, ^35^, ^36^ ^]^ Copyright 2019, Springer Nature; Copyright 2020, Springer Nature; Copyright 2021, Springer Nature; Copyright 2022, The American Association for the Advancement of Science. Copyright 2022, Springer Nature; Copyright 2023, Springer Nature; Copyright 2024, Springer Nature; Copyright 2025, Springer Nature; Copyright 2025, Springer Nature; Copyright 2025, John Wiley and Sons.

Over the past few years, the development of 2D ferroelectric semiconductor devices has undergone rapid and diverse evolution, marked by breakthroughs in architecture, mechanism, and functionality, as illustrated in **Figure** **1**. In 2019, the ferroelectric semiconductor field effect transistor (FeS‐FET) was first demonstrated using α‐In_2_Se_3_ to embed ferroelectricity within the channel, enabling direct carrier modulation without conventional gate stacks.^[^ ^27^ ^]^ This was followed in 2020 by enhanced tunneling electroresistance in CIPS‐based 2D ferroelectric tunnel junctions (FTJs), advancing logic‐in‐memory integration.^[^ ^28^ ^]^ In 2021, the adoption of MFMIS architectures improved retention and endurance by mitigating depolarization effects.^[^ ^29^ ^]^ From 2022 to 2023, novel mechanisms were uncovered, including spontaneous ferroelectricity in untwisted MoS_2_/WS_2_ heterostructures, tunable polarization in WSe_2_/MoS_2_ trilayers, and hybrid negative‐capacitance tunneling devices achieving sub‐60 mV dec^−1^ switching.^[^ ^30^, ^31^ ^]^ In 2024, a shear‐induced sliding ferroelectric transistor based on 3R‐MoS_2_ offered rewritable, high‐speed memory functionality.^[^ ^32^ ^]^ By 2025, the field expanded into optoelectronics, with demonstrations of ferroelectric bulk photovoltaic effects in 3R‐WS_2_^[^ ^33^ ^]^ and strong terahertz emission and synaptic plasticity in NbOI_2_^[^ ^34^ ^]^ heterostructures. Collectively, these advancements mark a transition from fundamental material exploration to multifunctional, device‐integrated platforms. 2D ferroelectric semiconductors are now emerging as key building blocks for next‐generation electronic, optoelectronic, and neuromorphic systems.

**Figure 2:**
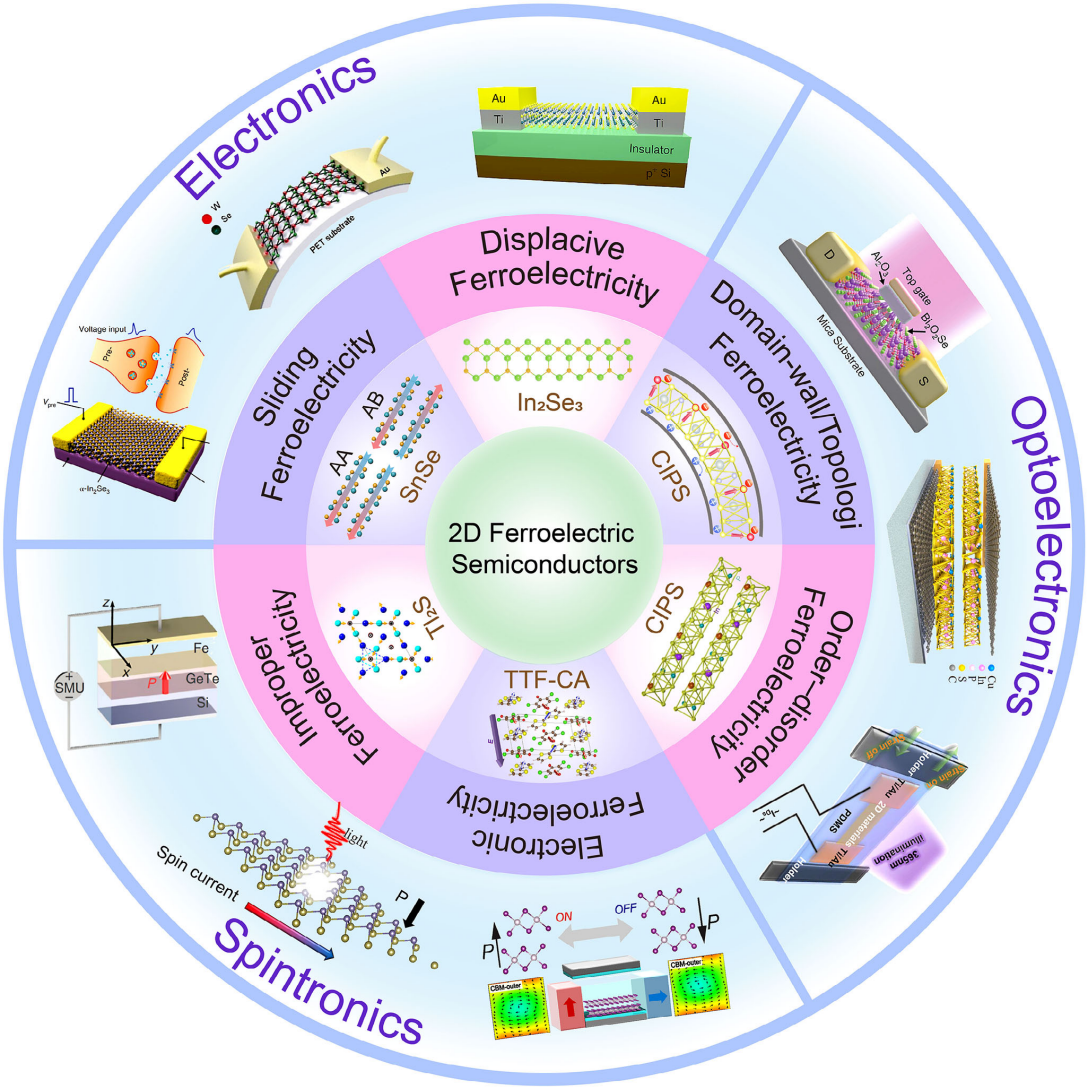
Progress in 2D ferroelectric semiconductors: structures, mechanisms, devices, and functional applications. The central circle highlights representative mechanisms of 2D ferroelectricity, including displacive ferroelectricity and sliding ferroelectricity,^[^ ^37^ ^]^ Copyright 2025, The Authors, published by Springer Nature. order–disorder ferroelectricity,^[^ ^13^ ^]^ Copyright 2016, The Authors, published by Springer Nature. improper ferroelectricity,^[^ ^38^ ^]^ electronic ferroelectricity,^[^ ^39^ ^]^ and domain‐wall topology ferroelectricity.^[^ ^40^ ^]^ The outer ring depicts potential applications in electronics,^[^ ^27^, ^41^, ^42^ ^]^ optoelectronics,^[^ ^25^, ^43^, ^44^ ^]^ references^25,^ ^44^, Copyright 2021, The Authors, published by Springer Nature. and spintronics.^[^ ^45^, ^46^, ^47^ ^]^ Reproduced with permission.^[^ ^27^, ^38^, ^39^, ^40^, ^41^, ^42^, ^43^, ^45^, ^46^, ^47^ ^]^ Copyright 2024, American Chemical Society; Copyright 2012, American Physical Society; Copyright 2024, American Chemical Society; Copyright 2019, Springer Nature; Copyright 2022, Springer Nature; Copyright 2017, John Wiley and Sons; Copyright 2023, John Wiley and Sons; Copyright 2021, Springer Nature; Copyright 2024, American Physical Society; Copyright 2024, American Chemical Society.

2D ferroelectric semiconductors form an emerging class of materials that combine spontaneous polarization with semiconducting behavior at atomic thickness. Materials such as α‐In_2_Se_3_ and CIPS exhibit stable room‐temperature ferroelectricity even in ultrathin films, offering promising opportunities for applications in electronics and optoelectronics. In this review, we first outline the historical context of ferroelectricity and the advent of 2D ferroelectrics, emphasizing the unique mechanisms that stabilize polarization at the nanoscale. It then discusses the fundamental principles that govern ferroelectricity in low‐dimensional systems, with a focus on their distinct physical properties compared to bulk ferroelectrics. Next, we outline the main classes of 2D ferroelectric semiconductors, including intrinsic materials and extrinsically engineered systems. Their structural features, polarization mechanisms, and representative examples are summarized in detail. In addition, applications in various device platforms are also discussed, including ferroelectric transistors, tunneling junctions, photodetectors, and spin‐related devices. The review concludes with an analysis of current challenges in the field, such as maintaining polarization stability at the nanoscale, integrating with existing semiconductor processes, and optimizing material performance. Future directions in materials discovery, theoretical modeling, and quantum ferroelectric effects are also considered. This review is intended to support researchers in advancing the development and application of 2D ferroelectric semiconductors. **Figure** **2** presents a schematic overview of the review structure, which helps to understand the overall organization and content arrangement of the article.

## 2 Fundamentals of 2D Ferroelectric Semiconductors

Understanding the fundamental physics of 2D ferroelectric semiconductors requires revisiting the core principles of ferroelectric ordering, which arises from the spontaneous alignment of electric dipoles within a crystal lattice. In conventional bulk and thin‐film systems, spontaneous dipole alignment is destabilized in ultrathin regimes due to enhanced depolarization fields and weakened Coulomb interactions, often leading to the disappearance of ferroelectricity below a few nanometers. In contrast, van der Waals layered materials present a fundamentally different platform. Their intrinsic 2D nature, with strong IP covalent bonding and weak out‐of‐plane (OOP) van der Waals coupling, offers structural stability down to the monolayer limit without the need for epitaxial constraint or chemical passivation. This anisotropic bonding environment suppresses detrimental interfacial effects and enables the retention of spontaneous polarization even in atomic‐scale systems. Beyond classical ferroelectric behavior, these 2D materials exhibit coupling between polarization and symmetry‐breaking electronic properties such as Rashba spin‐splitting, valley polarization, and direction‐dependent transport, making them ideal for exploring new regimes of ferroic order and correlated phenomena in reduced dimensions.

The microscopic mechanism of ferroelectric polarization is complex, as it typically involves competing interactions. Fundamentally, ferroelectricity originates from spontaneous symmetry breaking in the crystal structure under certain conditions, leading to spontaneous polarization. According to the soft mode theory,^[^ ^48^, ^49^ ^]^ ferroelectric phase transitions are generally driven by the softening of a specific optical phonon mode, whose frequency approaches zero, thereby inducing stable atomic displacements and forming a polar structure. This softening arises mainly from the competition between short‐range repulsive forces, which favor high‐symmetry nonpolar Jahn–Teller effect. Calculations of projector structures and long‐range Coulomb interactions, which stabilize low‐symmetry polar phases. In addition, the driving forces behind atomic displacements are often closely linked to electronic structure effects, including orbital hybridization,^[^ ^50^ ^]^ the projected density of states,^[^ ^50^ ^]^ interatomic force constants,^[^ ^51^ ^]^ and Born effective charges.^[^ ^52^ ^]^ These factors further clarify how changes in electronic structure drive lattice distortions and ultimately lead to ferroelectricity.

### 2.1 Origin of Ferroelectricity in 2D Materials

**Figure 3:**
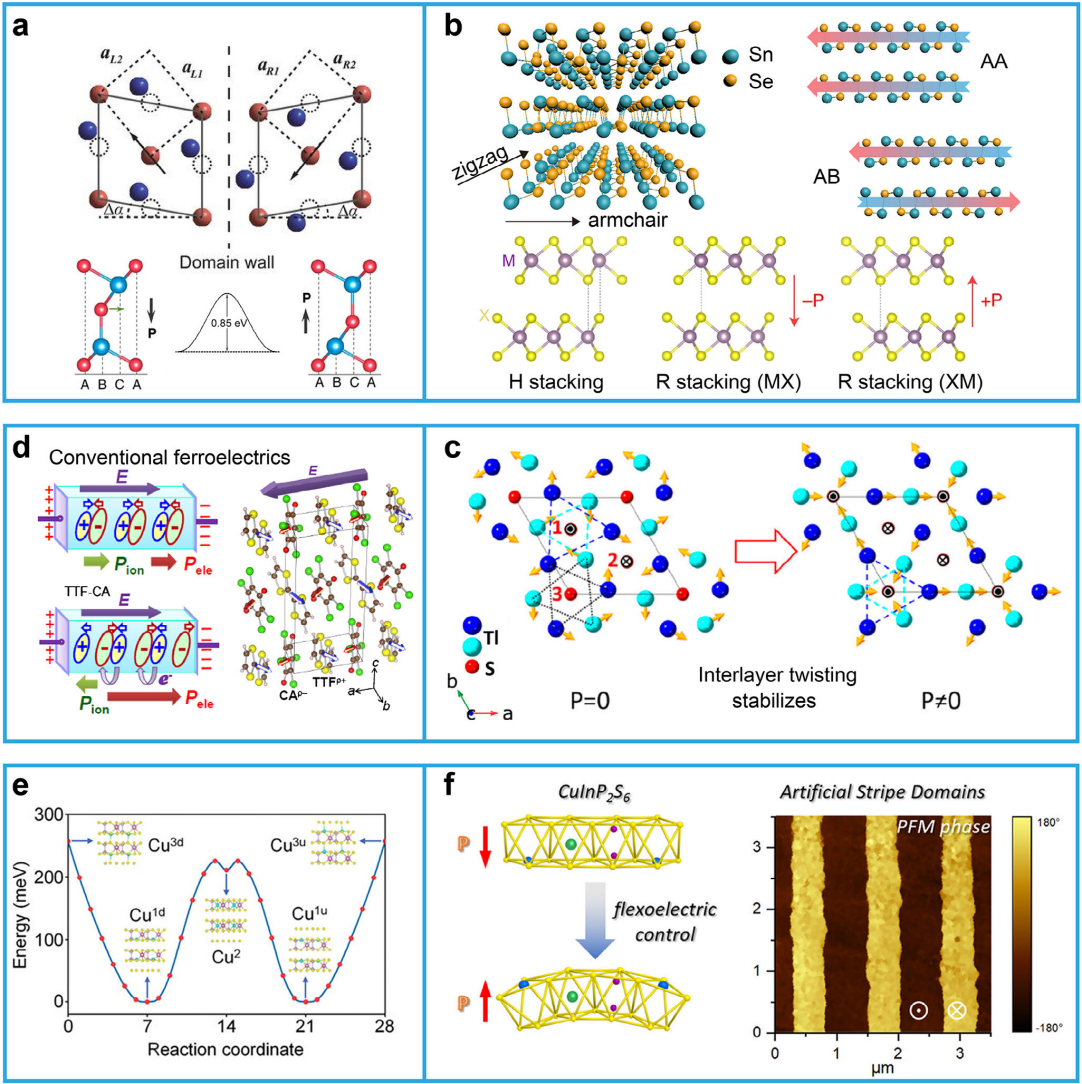
Representative mechanisms of 2D ferroelectricity. a) Displacive ferroelectricity in monolayer SnTe. The ferroelectricity originates from relative lateral displacements of Ge and Te atoms (top). Reproduced with permission.^[^ ^18^ ^]^ Copyright 2016, The American Association for the Advancement of Science. In In_2_Se_3_, the vertical displacement of Se atoms relative to In atoms breaks the inversion symmetry and induces spontaneous polarization (down).^[^ ^12^ ^]^ Copyright 2017, The Authors, published by Springer Nature. b) Sliding ferroelectricity in layered SnSe. Relative sliding between atomic layers (AA, AB, and different R stacking configurations) breaks centrosymmetry and induces polarization (top).^[^ ^37^ ^]^ Copyright 2015, The Authors, published by Springer Nature. In bilayer TMDs, H‐stacking with antiparallel layers restores inversion symmetry and gives zero net polarization, while r‐stacking with parallel MX or XM alignment induces OOP polarization (−P or +P) due to vertical atomic asymmetry (down). Reproduced with permission.^[^ ^60^ ^]^ Copyright 2022, Springer Nature. c) Improper ferroelectricity in Tl_2_S. Interlayer twisting stabilizes ferroelectricity by coupling structural instabilities with polar distortions, leading to P ≠ 0. Reproduced with permission.^[^ ^38^ ^]^ Copyright 2024, American Chemical Society. d) Electronic ferroelectricity in TTF‐CA. Polarization arises mainly from electronic charge redistribution, as revealed by the opposite direction of ionic and electronic contributions. Reproduced with permission.^[^ ^39^ ^]^ Copyright 2012, American Physical Society. e) Order–disorder ferroelectricity in CIPS. Cu⁺ ions switch between off‐center sites, resulting in a double‐well potential and ferroelectric ordering upon cooling. Reproduced with permission.^[^ ^71^ ^]^ Copyright 2023, John Wiley and Sons. f) Domain‐wall and flexoelectric‐induced ferroelectricity in CIPS. Flexoelectric control enables artificial stripe domains with local ferroelectricity, confirmed via piezoresponse force microscopy phase imaging. Reproduced with permission.^[^ ^73^ ^]^ Copyright 2022, American Chemical Society.

In intrinsic 2D ferroelectrics, polarization arises from the combined contributions of both electronic and ionic components. Based on symmetry‐breaking modes, the polarization mechanisms of 2D ferroelectric materials can be roughly classified into six types, as illustrated in **Figure** **3** and **Table** **1**. Most 2D ferroelectrics exhibit multiple polarization mechanisms simultaneously. For instance, the α‐phase In_2_Se_3_ displays multiple coexisting ferroelectric mechanisms. In In_2_Se_3_, the displacive ferroelectricity involves IP polarization induced by the lateral displacement of In and Se atoms, as well as OOP polarization arising from the vertical displacement of the central Se layer.^[^ ^12^, ^15^ ^]^ These two polarization directions coexist, leading to biaxial ferroelectricity.^[^ ^53^ ^]^ Some studies suggest that order–disorder‐like behavior may also be present during the phase transition from the high‐temperature β‐phase to the low‐temperature α‐phase, although the dominant mechanism remains ionic displacement. In multilayer In_2_Se_3_, sliding ferroelectricity arises from changes in stacking symmetry due to interlayer translation, allowing reversible switching between ferroelectric and antiferroelectric states.^[^ ^54^ ^]^

**Table 1:** Summary of Ferroelectric Mechanisms and Parameters in 2D ferroelectric semiconductors.

Mechanisms	Materials	Polarization direction	*T*_c_ [K]	*E*_c_	*P*	Refs.
Displacive	α‐In_2_Se_3_	OOP + IP	473	0.1–0.4 V nm^−1^	7–8 µC cm^−^^2^	[27]
	SnTe	IP	270	–	–	[18]
	GeTe	IP	570	–	–	[55]
	CuInP_2_S_6_	OOP	320	25–30 kV cm^−1^	3–5 µC cm^−^^2^	[77, 78, 79]
	SnS	IP	>300	–	1.8‐4.8 C m^−^¹	[11, 17]
Sliding	WTe_2_	OOP	350	–	0.051 µC cm^−^^2^	[56, 80]
	BN	OOP	620	0.3 V nm^−1^	0.68 µC cm^−^^2^	[58, 81]
	β‐InSe	OOP	>300	0.15 V nm^−1^	0.375 µC cm^−^^2^	[59, 80]
	MX_2_ (M = Mo,W; X = S,Se)	OOP	–	–	0.77 pC m^−1^ 0.59 pC m^−1^ 0.69 pC m^−1^ 0.73 pC m^−1^	[82]
	3R‐MoS_2_	OOP	>650	0.1–0.19 V nm^−1^	–	[83]
	SeSn	OOP				[37]
	GaSe	OOP			6.19 pC m^−1^	[84]
Improper	Tl_2_S	OOP	–	–	–	[38]
	MoTe_2_	OOP/IP	>300	–	–	[85]
	Twisted/Moiré h‐BN	OOP	>300	0.1 V nm^−1^	0.68 µC cm^−2^	[58]
	Bi_2_O_2_Se	OOP	508	–	4.4 pm V^−1^	[21, 43]
	d1T‐MoS_2_	OOP	–	–	–	[86]
	Hf_2_VC_2_F_2_	*c*‐axis	>300	–	0.27 µC cm^−^^2^	[87]
Electronic	TTF‐CA	*a*/*c*‐axis	71	–	6.3 µC cm^−^^2^	[39]
	Sc_2_CO_2_	OOP	300–600	2.5 V nm^−1^	1.6 µC cm^−^^2^	[88, 89]
	1T’‐WTe_2_	IP	350	–	0.2–0.4 µC cm^−^^2^	[56]
Order–disorder	CuInP_2_S_6_	OOP	333 K	200 kV cm^−1^	3–4 µC cm^−^^2^	[71]
	CuCrP_2_S_6_	OOP	305–320	–	14.97 µC cm^−^^2^	[16]
	CuBiP_2_Se_6_	OOP	–	–	–	[90]
Domain‐wall	WTe_2_	OOP	350	1–10 GV m^−1^	0.3–0.5 µC cm^−^^2^	[56, 91]
	Twisted/Moiré h‐BN	OOP	>300	0.1 V nm^−1^	0.68 µC cm^−2^	[58]
	SnSe	400	>300	–	–	[92]

#### 2.1.1 Displacive Ferroelectricity

Displacive ferroelectricity is one of the most common mechanisms underlying the emergence of ferroelectricity in materials, in which polarization originates from the displacement of ions or atoms away from their centrosymmetric positions within the crystal lattice. When the crystal structure undergoes a phase transition from a high‐symmetry (non‐polar) phase to a low‐symmetry (polar) phase, the central ions or atoms in the unit cell shift from their equilibrium positions. These displacements break the symmetry of the system and result in spontaneous polarization, where the positive and negative charges are separated, giving rise to a dipole moment at the atomic scale. The critical feature is that the polarization is directly related to the degree of ion displacement. In 2D materials like In_2_Se_3_,^[^ ^12^ ^]^ SnTe,^[^ ^18^ ^]^ and GeTe,^[^ ^8^, ^55^ ^]^ this mechanism is observed. In monolayer SnTe, spontaneous IP polarization arises from the relative displacement between Sn and Te atoms in its puckered lattice (Figure 3a, top). The FE stems of α‐In_2_Se_3_ from the vertical displacement of Se atoms in the center (Figure 3a, down). In monolayer GeTe, displacive ferroelectricity arises from a relative lateral displacement between Ge and Te atoms, which breaks the inversion symmetry of the lattice and induces strong IP spontaneous polarization. In its paraelectric phase, Ge and Te atoms are symmetrically arranged within a hexagonal lattice. Upon transitioning to the ferroelectric phase, Ge atoms shift off‐center relative to Te atoms, forming a polar structure. This distortion is driven by the softening of transverse optical phonon modes, a signature of displacive ferroelectricity, and leads to a characteristic double‐well potential energy surface. First‐principles calculations reveal that this displacement is stabilized by the competition between short‐range repulsion and long‐range Coulomb interactions, as well as the hybridization between Ge 4s and Te 5p orbitals, which contributes to the energy gain in the polar structure. Monolayer GeTe exhibits robust IP polarization (≈2–4 µC cm^−^^2^) and a high Curie temperature exceeding 500 K.^[^ ^11^ ^]^

#### 2.1.2 Sliding Ferroelectricity

Sliding ferroelectricity emerges in vdW layered materials, where relative sliding between adjacent atomic layers breaks inversion symmetry and generates spontaneous polarization. Unlike displacive ferroelectricity, where polarization originates from intralayer ionic displacements, sliding ferroelectricity is interlayer in nature, driven by the stacking arrangement and lateral translation of atomic planes. This mechanism is governed by weak interlayer vdW interactions, which allow for low‐energy sliding between layers. The polarization is often OOP and strongly dependent on the stacking configuration (e.g., AA, AB, BA). Sliding ferroelectricity is switchable via lateral electric fields or mechanical shear strain, which can shift one layer relative to another. The energy barrier for switching is often low due to the weak interlayer bonding, making this type of ferroelectricity promising for low‐power applications. In 2018, Fei et al. experimentally demonstrated sliding ferroelectricity for the first time in few‐layer WTe_2_.^[^ ^56^ ^]^ Subsequently, in 2019, Sharma et al. further confirmed the presence of OOP ferroelectricity in bulk WTe_2_ using piezoresponse force microscopy (PFM).^[^ ^57^ ^]^ In 2020, Yasuda et al. designed a dual‐gated van der Waals heterostructure device, in which monolayer graphene was used as a detector to quantitatively probe the polarization of bilayer BN.^[^ ^58^ ^]^ Sliding ferroelectricity has also been observed in semiconducting van der Waals layers. For instance, Hu et al. detected clear amplitude butterfly loops and phase hysteresis in ≈7 nm‐thick β‐InSe via PFM, revealing reversible OOP polarization switching.^[^ ^59^ ^]^ In 2021, Wang et al. identified sliding ferroelectricity in bilayers of a series of transition metal dichalcogenides (TMDs), including MoS_2_, WS_2_, MoSe_2_, and WSe_2_.^[^ ^60^ ^]^ By introducing AA stacking and interlayer sliding in SnSe, Chen et al. induced polarization and uncovered the coupling between OOP and IP polarization in SeSn,^[^ ^37^ ^]^ as shown in Figure 3b. Therefore, in van der Waals materials with centrosymmetric lattice structures, ferroelectricity can be achieved through interlayer sliding, exploiting the slip‐induced polarization mechanism.

#### 2.1.3 Moiré/Twistronic Ferroelectricity

Beyond sliding ferroelectricity, recent studies on moiré and twistronic systems have unveiled novel ferroelectric phenomena that greatly enrich the physical picture of 2D polarization. The so‐called moiré or twistronic ferroelectricity arises from long‐range moiré superlattice potential modulations induced by small twist angles (typically <2°) or lattice‐constant mismatches between adjacent layers.^[^ ^58^, ^61^, ^62^ ^]^ Such periodic potential wells lead to spatial modulation of both electronic states and atomic configurations, resulting in the emergence of polarized domains, soliton lattices, and domain‐wall network structures in local regions.^[^ ^63^ ^]^ When the twist angle approaches a critical small value (<1°), pronounced atomic reconstruction occurs, breaking the original crystalline symmetry and inducing spontaneous polarization in 2D van der Waals bilayers.^[^ ^64^ ^]^ A typical example is the twisted bilayer hexagonal boron nitride (tBL BN), where slight misalignment and reconstruction of local ionic dipoles between boron and nitrogen atoms under small twist angles induce O polarization and distinct ferroelectric hysteresis behavior.^[^ ^58^, ^65^, ^66^ ^]^ In TMD semiconductors, such as twisted bilayer WSe_2_, MoSe_2_, WS_2_, and MoS_2_, small‐angle twisting generates triangular moiré domain patterns composed of MX and XM stacking regions, separated by domain walls carrying fractionalized polarization charges.^[^ ^60^ ^]^ Polarization reversal typically proceeds through the motion of domain walls and collective rearrangement of the soliton network, with the associated energy barriers being tunable via twist angle,^[^ ^67^ ^]^ stacking configuration,^[^ ^60^ ^]^ and external electric field.^[^ ^64^ ^]^ Compared with sliding ferroelectricity, moiré ferroelectricity features a fundamentally distinct energy landscape and switching mechanism. The former is dominated by interlayer translation with low energy cost, while the latter involves cooperative reconstruction of soliton domains and domain‐wall networks, exhibiting enhanced tunability and strong responsiveness to external fields. This unique programmable polarization behavior offers a new platform for the design of reconfigurable electronic devices, nonvolatile optoelectronic modulators, and quantum information units based on polar solitons.

#### 2.1.4 Improper Ferroelectricity

In improper ferroelectrics, polarization is not the primary order parameter but instead emerges as a secondary effect induced by a nonpolar structural distortion, such as rotational, tilt, or lattice modulation modes.^[^ ^68^ ^]^ This is fundamentally different from proper ferroelectricity, where spontaneous polarization arises directly from a polar instability (e.g., a soft optical phonon mode). In improper ferroelectrics, the polar distortion is driven or stabilized by other nonpolar distortions through anharmonic coupling, typically in the form of trilinear or higher‐order coupling terms in the free energy. Improper ferroelectricity is typically found in hybrid improper ferroelectrics, multiferroics, and layered perovskites. Gui et al., based on first‐principles calculations, revealed an improper, electronically driven ferroelectric mechanism in Tl_2_S induced by structural instability. This improper ferroelectricity originates from an unstable phonon mode (K_2_a) at the Brillouin zone boundary K point (1/3, 1/3, 0), which nonlinearly couples with the intrinsic polar mode (*Γ*_2−_) to induce OOP polarization^[^ ^38^ ^]^ (Figure 3c). Unlike conventional ion‐displacement‐driven ferroelectricity, the resulting polarization arises primarily from electronic redistribution, exhibiting characteristic features of electronic improper ferroelectricity. CIPS is commonly classified as a proper ferroelectric, with its ferroelectricity primarily originating from the displacement of Cu⁺ ions along the OOP direction. While this mechanism is characteristic of proper ferroelectricity, recent studies have explored the complex interplay of structural distortions in CIPS. For instance, the coexistence of ferrielectric states and non‐piezoelectric surface phases has been observed, suggesting that under certain conditions,^[^ ^69^ ^]^ polarization in CIPS may be influenced by secondary structural modes, hinting at improper ferroelectric behavior.

#### 2.1.5 Electronic Ferroelectricity

Electronic ferroelectricity is characterized by spontaneous polarization that arises predominantly from the redistribution of electronic charge, rather than from ionic displacements. In such systems, a shift in the electronic charge center relative to the ionic lattice generates a net electric dipole, even when atomic positions remain essentially unchanged. This mechanism is distinct from conventional displacive ferroelectricity, where polarization results from symmetry‐breaking ionic movements. The origin of polarization in electronic ferroelectrics typically lies in changes to the electronic band structure, charge density rearrangement, or orbital hybridization. Due to the ultrafast response of electronic degrees of freedom, this type of ferroelectricity holds promise for high‐speed electronic and optoelectronic devices. Electronic ferroelectricity is an intrinsic polarization mechanism driven by electronic behavior itself and is commonly found in conjugated organic molecules or certain 2D resonant systems. TTF‐CA (tetrathiafulvalene–p‐chloranil), despite being 1D, is one of the earliest and most representative materials experimentally confirmed to exhibit electronic ferroelectricity. Kobayashi et al., using Berry phase theory, demonstrated that the polarization direction is opposite to that of ionic displacement, with the ionic contribution being negative and the electronic part dominating the total polarization,^[^ ^39^ ^]^ as shown in Figure 3d. This confirms that TTF‐CA is a prototypical electronic ferroelectric. Monolayer WTe_2_ is a representative example of this behavior in 2D systems. WTe_2_ exhibits switchable IP polarization driven by an asymmetric distribution of electron density across the layer. This asymmetry arises from the material's intrinsic lack of inversion symmetry and strong spin–orbit interaction. Experimental and theoretical studies have shown that the polarization can be reversed by an applied IP electric field,^[^ ^56^, ^70^ ^]^ with negligible ionic displacement involved. This confirms the electronic origin of the polarization.

#### 2.1.6 Order–Disorder Ferroelectricity

Order–disorder ferroelectricity arises from the thermally driven reorientation of local dipoles associated with discrete atomic configurations. In the paraelectric phase, these dipoles are dynamically disordered due to thermal fluctuations, leading to a vanishing net polarization. Upon cooling, the system undergoes a phase transition in which the dipoles align in a preferred orientation, resulting in spontaneous polarization. This mechanism contrasts with displacive ferroelectricity, where polarization emerges from continuous ionic displacements. A representative example in 2D van der Waals materials is CIPS.^[^ ^71^ ^]^ In this compound, ferroelectricity originates from the off‐center displacement of Cu⁺ ions within sulfur octahedra (Figure 3e). At high temperatures, Cu⁺ ions reside at centrosymmetric sites, and the system becomes nonpolar. As temperature decreases, the ions shift into energetically favorable off‐center sites, leading to an ordered arrangement of local dipoles and a net polarization. Experimental techniques such as piezoresponse force microscopy and second‐harmonic generation, along with first‐principles calculations, have confirmed the order–disorder nature of this ferroelectric transition.

#### 2.1.7 Domain‐Wall/Topological Ferroelectricity

Domain‐wall ferroelectricity and topological ferroelectricity differ from the intrinsic polarization mechanisms discussed above, as they do not arise from the material's bulk structural polarity. Instead, the polarization in these cases is localized and non‐intrinsic. In certain 2D materials that are globally non‐ferroelectric or weakly ferroelectric, local symmetry breaking at domain walls can induce spontaneous polarization, resulting in localized ferroelectric behavior. Strain is an effective approach to induce ferroelectricity at domain walls. In In_2_Se_3_, local polarization can be generated through bending or localized stress, resulting in ferroelectric domains of varying sizes.^[^ ^72^ ^]^ In 2D CIPS, large‐scale stripe‐like ferroelectric domains can be formed via the flexoelectric effect, enabling mechanical control of polarization,^[^ ^73^ ^]^ as shown in Figure 3f. In bilayer MoTe_2_^[^ ^74^ ^]^ and WTe_2_,^[^ ^56^ ^]^ the polarization direction can be modulated by the position of domain walls. Topological ferroelectricity refers to the formation of ferroelectric domain configurations with topological features, such as vortices, skyrmions, or loop structures. These topological polar structures often exhibit enhanced stability and tunability, and are typically observed in hexagonal ferroelectrics or systems with strong domain coupling. Monolayer WTe_2_ is regarded as a representative 2D material exhibiting topological ferroelectricity.^[^ ^56^ ^]^ Its ferroelectricity arises from the reconstruction of orbital electronic states following lattice symmetry breaking, even in the absence of significant ionic displacement. The spontaneous polarization originates from anomalous orbital currents and Berry curvature, indicating an electronic topological origin of the ferroelectric behavior.^[^ ^75^, ^76^ ^]^

### 2.2 Unique Properties of 2D Ferroelectric Semiconductor

#### 2.2.1 Coexistence of Ferroelectricity and Semiconducting Behavior

2D ferroelectric semiconductors exhibit unique physical properties not found in conventional ferroelectric materials, particularly the coexistence of spontaneous polarization and semiconducting behavior. While traditional ferroelectrics such as BaTiO_3_ and PbTiO_3_ are typically wide‐bandgap insulators with limited charge transport capabilities, 2D materials like In_2_Se_3_, SnTe, SnS, 1T‐MoTe_2_, and Bi_2_O_2_Se combine robust ferroelectricity with moderate,^[^ ^93^ ^]^ tunable band gaps in the range of approximately one to two electronvolts. In these systems, polarization can directly modulate carrier distribution and band alignment via the internal electric field, enabling control over conduction type and allowing for self‐powered device operation without external gate voltages. Their atomic‐scale thickness and clean surfaces minimize leakage current, a major limitation in bulk ferroelectric oxides. This intrinsic coupling between polarization and semiconducting properties provides a versatile platform for multifunctional, low‐power, high‐density electronic and optoelectronic devices. Additionally, ferroelectric states in these materials can be effectively tuned through external strain,^[^ ^15^, ^94^, ^95^, ^96^ ^]^ offering further opportunities for precise control over their electronic and optical functionalities.

#### 2.2.2 Low Switching Barrier and Single‐Layer Ferroelectric Scalability

In 3D ferroelectrics, OOP polarization is typically suppressed due to depolarization effects, which limit the stability of ferroelectricity at ultrathin dimensions. For instance, in BaTiO_3_ thin films, ferroelectric polarization vanishes when the thickness falls below six unit cells.^[^ ^97^ ^]^ Although some conventional ferroelectrics can maintain polarization at reduced thicknesses, such as 2.4 nm for BaTiO_3_,^[^ ^97^ ^]^ 1.2 nm for PbTiO_3_,^[^ ^98^ ^]^ one unit cell for BiFeO_3_,^[^ ^99^ ^]^ and 1.5 unit cells for PbZr_0_._2_Ti_0_._8_O_3_,^[^ ^100^ ^]^ this typically requires carefully engineered ferroelectric–electrode interfaces with sufficient carrier densities to screen depolarizing fields or the application of compressive strain from the substrate to stabilize stripe domain structures. In contrast, 2D vdW materials can be exfoliated down to the monolayer limit without dangling bonds, effectively overcoming thickness‐related limitations. According to Li et al., monolayer SnTe exhibits stable IP ferroelectricity with a transition temperature of 270 K, attributed to enhanced quantum confinement and increased in‐plane lattice distortion.^[^ ^18^ ^]^ Biswas et al. reported that ultrathin Bi_2_O_2_Se nanoflakes exhibit robust OOP ferroelectricity at room temperature, driven by broken inversion symmetry caused by orthorhombic distortion.^[^ ^101^ ^]^ Yuan et al. found that monolayer 1T‐MoTe_2_ displays OOP ferroelectricity at room temperature, with a transition temperature exceeding 330 K.^[^ ^20^ ^]^ Moreover, at atomic‐scale thickness, the energy barrier for polarization switching is reduced, enabling polarization control under low voltages using vertical or lateral electric fields. The thermal stability, hysteresis behavior, and switching speed of the polarization state in 2D structures can also be tuned through layer number, strain, or interfacial engineering.

#### 2.2.3 Negative Piezoelectric Coefficients

The negative piezoelectric coefficient, especially the negative longitudinal piezoelectric effect (NLPE), describes an atypical electromechanical behavior where polarization increases under compressive strain along the polarization axis. This contrasts with the conventional effect, where polarization typically decreases. NLPE is frequently observed in 2D vdW layered materials due to their structural anisotropy and unique interlayer interactions. The piezoelectric response includes two components: the clamped‐ion term, reflecting polarization change with fixed atomic positions, and the internal‐strain term, arising from atomic relaxations. A dominant negative contribution from either term results in a negative overall response. In Sb_2_TeSe_2_,^[^ ^102^ ^]^ both terms are strongly negative, yielding a longitudinal piezoelectric coefficient of ≈−32.666 pC N^−1^, among the highest known in 2D materials. In multilayer vdW ferroelectrics with OOP polarization, polarization is mainly confined within atomic layers, while interlayer forces are governed by vdW interactions. When an electric field enhances intralayer dipoles, interlayer dipole–dipole repulsion increases, causing vdW gaps to contract. This contraction can outweigh in‐plane lattice expansion, reducing the lattice constant along the polarization direction. In CIPS,^[^ ^103^, ^104^, ^105^ ^]^ highly mobile Cu ions migrate within layers and across vdW gaps, forming a quadruple‐well potential along the *z‐*axis with two energy minima per polarization direction. Intralayer migration corresponds to a low‐polarization state (±4.93 µC cm^−^^2^) that is strain‐sensitive, leading to a negative longitudinal piezoelectric coefficient. Migration into vdW gaps yields a high‐polarization state (±11.26 µC cm^−^^2^) with a positive response.

## 3 Classes of 2D Ferroelectric Semiconductors

2D ferroelectric semiconductors have garnered increasing attention due to their unique integration of switchable polarization and semiconducting behavior, enabling novel functionalities in logic, memory, sensing, and optoelectronic applications. A variety of 2D ferroelectric materials have been reported, and although the origin of ferroelectricity in some cases remains under debate, numerous theoretical studies have offered compelling explanations. Based on the origin of their ferroelectric properties, these materials can be broadly categorized into intrinsic and extrinsic types. Intrinsic 2D ferroelectric semiconductors exhibit spontaneous polarization as a direct consequence of their crystal symmetry and atomic configuration, even down to the monolayer limit. In contrast, extrinsic 2D ferroelectric semiconductors acquire ferroelectricity through external modifications such as interfacial coupling in layered heterostructures, strain engineering, surface functionalization, or defect engineering. This section provides an overview of both classes, with a focus on representative materials and the fundamental mechanisms underlying their ferroelectric behavior.

### 3.1 Intrinsic 2D Ferroelectric Semiconductors

Intrinsic 2D ferroelectric semiconductors exhibit spontaneous polarization arising directly from their low‐symmetry crystal structures and specific atomic arrangements, without the need for external stimuli, and they retain ferroelectric properties even at the monolayer limit. In TMDs, numerous ferroelectric semiconductors and ferroelectric metals have been identified, with semiconducting behavior typically associated with the 2H phase (e.g., MoS_2_, WS_2_, MoSe_2_, and WSe_2_) and metallic or semimetallic behavior more common in the 1T and 1T' phases (e.g., 1T‐MoS_2_, 1T'‐WTe_2_). Bismuth oxychalcogenides such as Bi_2_O_2_Se, Bi_2_O_2_Te, and Bi_2_O_2_S represent another class of 2D van der Waals ferroelectric semiconductors that combine high carrier mobility with intrinsic ferroelectricity. Group‐IV monochalcogenides, including SnS, SnSe, GeS, GeSe, SiS, and SiSe, have also been reported to exhibit ferroelectricity in their monolayer forms, with some studies indicating potential multiferroic behavior.

Among 2D ferroelectric semiconductor materials, In_2_Se_3_ is a prototypical ferroelectric semiconductor, exhibiting both IP and OOP ferroelectricity due to selenium atom displacement relative to the indium sublattice; its monolayer α‐phase breaks inversion symmetry and allows for electrically switchable polarization. Another well‐known material is SnTe, a group‐IV elemental material with a buckled lattice structure, which shows strong in‐plane ferroelectricity driven by spontaneous lattice distortion, and its dipole moment can be reversibly switched under applied bias. CIPS, a wide‐bandgap semiconductor (2.6–3 eV), displays ferroelectricity due to the relative displacement of cations and anions within its ionic framework, leading to vertical polarization. These intrinsic ferroelectric semiconductors not only provide model systems for exploring polarization switching mechanisms at the atomic scale but also offer significant potential for integration into low‐power, non‐volatile electronic devices.

### 3.2 Extrinsic 2D Ferroelectric Semiconductors

Engineered 2D ferroelectric semiconductors refer to 2D semiconductors that acquire ferroelectric properties through external modulation strategies such as interfacial engineering, strain, electric fields, twist angles, doping, or magnetic ordering. These materials are not intrinsically ferroelectric, but their symmetry and electronic structure are engineered to break inversion symmetry and induce a stable polarization.

In ferroelectric–semiconductor heterostructures, the electronic properties of the semiconductor layer can be directly modulated by altering the interfacial polarization charges via altering the polarization state of the ferroelectric layer, thereby enabling non‐volatile ferroelectric functionality within the semiconductor layer. A series of ferroelectric–semiconductor coupled systems,^[^ ^79^, ^94^, ^106^, ^107^ ^]^ such as PMN‐PT/MoS_2_, CIPS/MoS_2_, and In_2_Se_3_/MoS_2_, have been developed, in which the coexistence of semiconductor and ferroelectric properties and a strong coupling between semiconductor and ferroelectricity are observed. Moreover, the interfacial interaction between MoS_2_ and various substrates can also generate a vertical electric field at the interface, whose magnitude can be further tuned optically.^[^ ^108^ ^]^ In a related study, Weston et al. realized interfacial ferroelectricity in slightly twisted bilayer MoS_2_ by forming distinct stacking configurations (e.g., MotSb and StMob), where interlayer charge transfer induces spontaneous polarization.^[^ ^61^ ^]^ In stacked vdW devices, aligning all polarization directions requires substantial energy to bend and merge adjacent domain walls. Studies have demonstrated that near‐field infrared nanoimaging and nanophotocurrent techniques can visualize moiré ferroelectricity in graphene/twisted‐WSe_2_ heterostructures, enabling its investigation at the native length scale.^[^ ^65^ ^]^

The Janus structure provides an effective approach for constructing ferroelectric‐like materials, in which polarization originates from the chemical asymmetry of the crystal lattice. Owing to the different anions on the top and bottom layers (e.g., the electronegativity difference between S and Se in MoSSe), the electron density becomes unevenly distributed along the OOP direction, resulting in a permanent dipole moment.^[^ ^109^ ^]^ This dipole is fixed within the crystal lattice and cannot be reversed by an external electric field, thereby corresponding to a non‐intrinsic polarization rather than genuine ferroelectricity. Under an applied vertical electric field, however, the electronic charge distribution can be modulated, leading to a so‐called pseudo‐polarization switching. In addition, applying tensile or compressive strain can modify the Mo─X bond length and interlayer spacing, making the two polarization states nearly degenerate. For example, in Janus MoSSe, the vertical dipole moment increases from ≈360 e µm at –4% strain to 400 e µm at +4% strain.^[^ ^110^ ^]^ When Janus monolayers are integrated with other layered materials to form heterostructures, interfacial polarization reversal may occur, giving rise to ferroelectric‐like coupling behavior.^[^ ^111^ ^]^ It should be emphasized that these modulation processes do not represent genuine ferroelectric switching, but rather correspond to field‐induced redistribution of dipoles. Moreover, bilayer Janus‐type TMDs can combine with sliding or twistronic ferroelectricity.^[^ ^112^ ^]^ Theoretical calculations suggest that interlayer sliding in bilayer MoSSe induces an asymmetric charge redistribution between the upper and lower layers, enabling switchable polarization components both IP and OOP.

**Figure 4:**
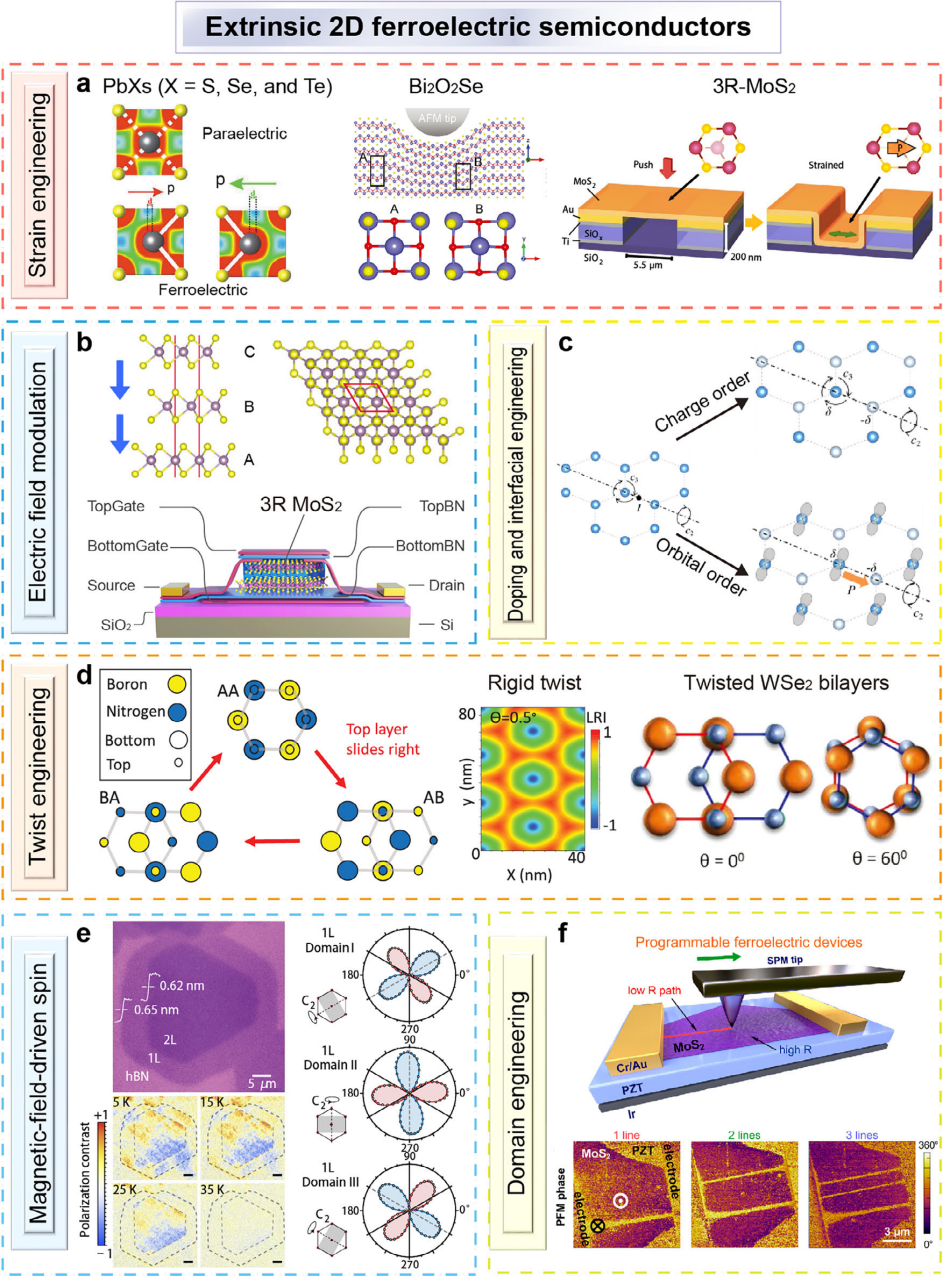
Extrinsic 2D ferroelectric semiconductors. a) Strain‐induced ferroelectric behavior. Reproduced with permission.^[^ ^120^, ^121^, ^122^ ^]^ Copyright 2020, John Wiley and Sons; Copyright 2023, John Wiley and Sons; Copyright 2023, Springer Nature. b) Electric field‐driven polarization switching. Reproduced with permission.^[^ ^83^ ^]^ Copyright 2022, Springer Nature. c) Doping and interface engineering. Reproduced with permission.^[^ ^123^ ^]^ Copyright 2018, American Physical Society. d) Twisting‐induced ferroelectricity. Reproduced with permission.^[^ ^124^, ^125^ ^]^ Copyright 2021, The American Association for the Advancement of Science. Copyright 2024, John Wiley and Sons. e) Magnetic‐field‐driven spin texture‐induced ferroelectricity. Reproduced with permission.^[^ ^14^ ^]^ Copyright 2022, Springer Nature. f) Domain engineering for local polarization control. Reproduced with permission.^[^ ^126^ ^]^ Copyright 2019, American Chemical Society.

Ferroelectricity can also be induced in semiconductors through the employed approach of strain engineering. When a semiconductor is subjected to non‐uniform strain or stress, a flexoelectric polarization field may emerge due to the flexoelectric effect,^[^ ^113^, ^114^, ^115^ ^]^ allowing for ferroelectric behavior in intrinsically non‐ferroelectric materials.^[^ ^116^, ^117^ ^]^ For instance, Guo et al. applied non‐uniform strain to Complementary Metal–Oxide–Semiconductor (CMOS) technology‐compatible silicon devices and successfully induced a polarization field via flexoelectric engineering.^[^ ^113^, ^118^ ^]^ In suspended MoS_2_ structures, a highly localized polarization field was generated by introducing a strain gradient using the tip of an atomic force microscope to create nonlinear bending deformations.^[^ ^119^ ^]^ Similarly, uniform strain applied to PbXs (*X* = S, Se, and Te),^[^ ^120^ ^]^ Bi_2_O_2_Se,^[^ ^121^ ^]^ and 3R‐MoS_2_^[^ ^122^ ^]^ has been shown to effectively induce ferroelectricity in these semiconductors (**Figure** **4**a).

Electric field modulation represents another effective approach. For example, applying a vertical electric field to multilayer (more than bilayer) 3R MoS_2_ can drive interlayer sliding, thereby breaking inversion symmetry and inducing polarization^[^ ^83^ ^]^ (Figure 4b). Doping is also capable of inducing ferroelectric polarization in semiconductors. In monolayer CrBr_3_, electron doping breaks orbital degeneracy and results in IP polarization through charge and orbital ordering^[^ ^123^ ^]^ (Figure 4c). Twist engineering, achieved by adjusting the interlayer twist angle or lateral displacement, offers a similar mechanism^[^ ^124^ ^]^ (Figure 4d). In bilayer WSe_2_, spontaneous ferroelectric polarization emerges at low temperatures when the twist angle is small.^[^ ^125^ ^]^ In type‐II multiferroic systems, spin textures driven by magnetic fields can give rise to ferroelectric polarization via magnetoelectric coupling. For example, in monolayer NiI_2_, a spin‐helix order with inherent chirality breaks spatial inversion symmetry, thereby inducing a finite polarization^[^ ^14^ ^]^ (Figure 4e). Finally, domain engineering provides a pathway for localized control of polarization. By applying an electrical bias using a conductive scanning probe, local polarization switching has been demonstrated in MoS_2_/ferroelectric oxide heterostructures^[^ ^126^ ^]^ (Figure 4f).

## 4 Device Applications

**Figure 5:**
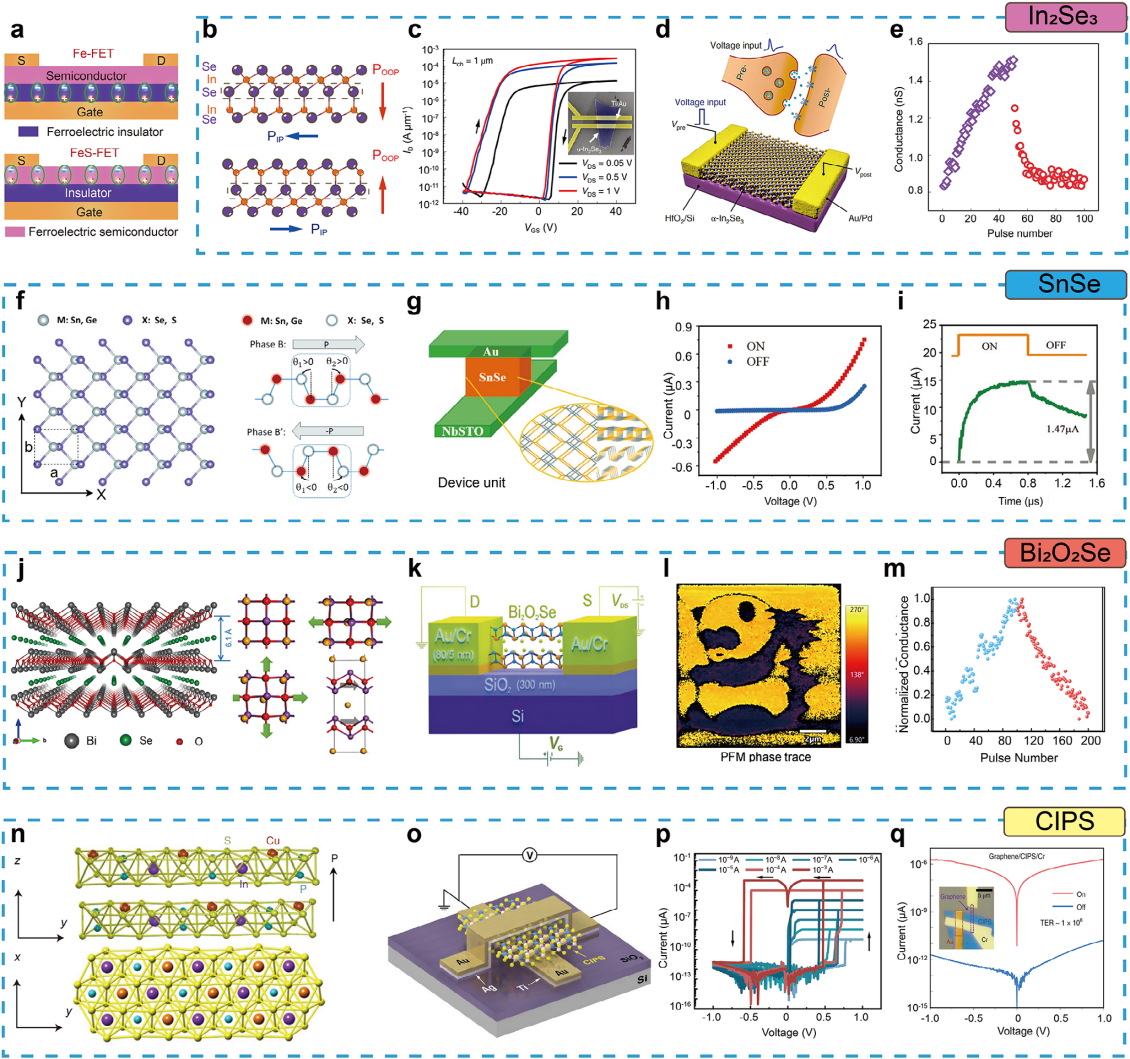
Applications of ferroelectric semiconductors in electronics. a) Schematic diagrams of ferroelectric transistors and ferroelectric semiconductor transistors. b) Lattice structures of In_2_Se_3_ with different polarization orientations. c) *I*_D_–*V*_DS_ characteristics at room temperature of a typical α‐In_2_Se_3_ FeS‐FET with 90 nm SiO_2_ gate dielectric and ALD passivation, featuring a 1 µm channel length and 52.2 nm thickness. Reproduced with permission.^[^ ^27^ ^]^ Copyright 2019, Springer Nature. d) α‐In_2_Se_3_ electronic synapse schematic. e) Long‐term potentiation by 50 pulses (2 V, 100 ms) and long‐term depression by 50 pulses (−2 V, 10 ms), with a −0.1 V bias for state readout. Reproduced with permission.^[^ ^42^ ^]^ Copyright 2022, Springer Nature. f) Top view of monolayer group‐IV monochalcogenide structure (left). Schematic side views of two distorted degenerate polar structures (B and B′) (right). Reproduced with permission.^[^ ^11^ ^]^ Copyright 2016, American Physical Society. g) Schematic of the Au/SnSe/NSTO memristor structure and 2D SnSe. h) Current of the SnSe device in ON and OFF states under low bias. i) The device current stabilizes after an 800 ns pulse with a Δ*I* of ≈1.47 µA. Reproduced with permission.^[^ ^144^ ^]^ Copyright 2020, RCS Pub. j) Layered crystal structure of Bi_2_O_2_Se and its lattice structure under strain. Reproduced with permission.^[^ ^21^, ^146^ ^]^ Copyright 2019, American Chemical Society; Copyright 2017, American Chemical Society. k) Back‐gate FETs based on Bi_2_O_2_Se thin films. l) PFM phase images of Bi_2_O_2_Se after Panda pattern writing with ±10 V DC voltage. Reproduced with permission.^[^ ^147^ ^]^ Copyright 2024, John Wiley and Sons. m) Normalized conductance states of the Bi_2_O_2_Se memristor versus the number of applied pulses. Reproduced with permission.^[^ ^148^ ^]^ Copyright 2017, American Chemical Society. n) CIPS crystal structure.^[^ ^13^ ^]^ Copyright 2016, The Authors, published by Springer Nature. o) Schematic of the ClPS resistive switching device on SiO_2_/Si substrate. p) Typical *I*–*V* curves of the Ag/ClPS/Au device under different constant current conditions. Reproduced with permission.^[^ ^135^ ^]^ Copyright 2023, Springer Nature. q) *I*–*V* characteristics of the ON and OFF states for bilayer graphene/CIPS/Cr heterostructures. Reproduced with permission.^[^ ^28^ ^]^ Copyright 2020, Springer Nature.

In recent years, the rapid progress of 2D ferroelectric semiconductors has given rise to novel device architectures distinct from conventional ferroelectric insulator structures, as shown in **Figure** **5**a. **Table** **2** summarizes the key parameters of representative 2D ferroelectric semiconductor devices and traditional ferroelectric devices. In conventional ferroelectric devices such as ferroelectric field‐effect transistors (FeFETs), a ferroelectric insulator is used as the gate dielectric to modulate the carrier density in the semiconductor channel. This approach often suffers from issues such as depolarization fields, charge trapping, interface defects, and gate leakage, which limit device performance and reliability. In contrast, 2D ferroelectric semiconductors combine ferroelectricity and semiconducting properties in a single material, enabling their direct use as the channel. Polarization switching takes place within the channel itself, not in the gate dielectric. Mobile carriers in the channel can screen depolarization fields, improving polarization stability. The clean, dangling‐bond‐free surfaces of 2D materials help reduce interface defects. This structure also allows for high on/off ratios and lower power consumption. These characteristics make 2D ferroelectric semiconductors a promising platform for next‐generation, highly integrated electronic systems. This section highlights recent advances in related devices, including applications in electronics, optoelectronics, and spintronics.

**Table 2:** Representative 2D ferroelectric devices and their key performance metrics.

Device type	Material system	On/off ratio	Memory window	Operating voltage	Retention	Endurance	Refs.
FeS‐FET	α‐In_2_Se_3_	≈10^8^	≈4 V	±6 V	–	–	[27]
	α‐In_2_Se_3_	≈10^5^	≈13 V	±10 V	–	>10^2^ cycles	[127]
	α‐In_2_Se_3_	≈10^5^	≈5 V	±8 V	>10^2 ^s	>10^2^ cycles	[24]
	α‐In_2_Se_3_	≈10^5^	≈3 V	±5 V	>10^3 ^s	>10^3^ cycles	[128]
	Bi_2_O_2_Se	≈10^4^	20 V	±50 V	–	–	[129]
FeFET	CIPS	10^7^	≈120 V	±80 V	10^4 ^s	300 cycles	[130]
	CIPS	10^6^	≈15 V	±10 V	10^4 ^s	10^4^ cycles	[131]
FSJ	α‐In_2_Se_3_	>10^4^	–	–	>10^4 ^s	>10^6^ cycles	[132]
	SnS	>20	–	–	≈10^3 ^s	10^4^ cycles	[133]
FTJ	CIPS	TER ≈ 10^6^	–	–	>10^4 ^s	>10^4^ cycles	[28]
Memristor	Bi_2_O_2_Se	10^10^	–	–	10^4 ^s	>10^2^ cycles	[134]
	CIPS	10^3^	–	–	10^4 ^s	>10^2^ cycles	[135]
FeFET	P(VDF‐TrFE)	10^4^	≈2 V	±6 V	10^4 ^s	10^2^ cycles	[136]
	HZO	10^5^	–	±1.5 V	10^8 ^s	10^4^ cycles	[137]
	PZT	10^7^	≈2 V	±3.5 V	>10^4 ^s	>10^2^ cycles	[138]
FeS	Advantage	High retention and low charge trapping					
	Disadvantage	Low polarization and low industrial applicability					
Traditional Fe	Advantage	high polarization and high industrial applicability					
	Disadvantage	Bulk scale layer and environmental issues					

### 4.1 Electronics

2D ferroelectric semiconductors integrate spontaneous polarization with intrinsic semiconducting behavior, providing an ideal material platform for developing nonvolatile memories, logic transistors, and neuromorphic devices. Their atomic‐scale thickness allows efficient electrostatic coupling and facile polarization modulation in vdW heterostructures. Representative 2D FeSCs, including α‐In_2_Se_3_, group‐IV monochalcogenides (MX, M = Ge, Sn; X = S, Se, and Te), Bi_2_O_2_Se, and CIPS, have demonstrated remarkable ferroelectricity and device functionalities.

#### 4.1.1 α‐In_2_Se_3_‐Based Devices

In_2_Se_3_ is a prototypical 2D van der Waals ferroelectric semiconductor, with its monolayer structure composed of a Se─In─Se─In─Se atomic stacking sequence (Figure 5b). Among its five phases (*α*, *β*, *γ*, *δ*, and *κ*), the *α* phase is the most stable and exhibits coupledIP and OOP polarization in both 2H and 3R stacking configurations. In 2019, Si et al. developed an α‐In_2_Se_3_‐based FeS‐FET exhibiting an on/off ratio over 10⁸, an on‐state current of 862 µA µ^−1^ m^−1^, low operating voltage, and a wide memory window, demonstrating its potential for low‐power non‐volatile memory,^[^ ^27^ ^]^ as shown in Figure 5c. Beyond logic functions, α‐In_2_Se_3_ has shown promising neuromorphic capabilities. Under electrical stimulation, it exhibits controllable temporal dynamics (Figure 5d). As shown in Figure 5e, synaptic devices based on α‐In_2_Se_3_ are capable of long‐term conductance modulation, including long‐term potentiation (LTP) and long‐term depression (LTD), achieved by applying multiple identical voltage pulses that induce gradual ferroelectric polarization switching.^[^ ^42^ ^]^ These properties highlight the potential of α‐In_2_Se_3_‐based synaptic devices for brain‐inspired computing. In FTJs architectures, α‐In_2_Se_3_ also serves as an efficient ferroelectric barrier layer. Si et al. (2021) introduced α‐In_2_Se_3_ with asymmetric electrodes, achieving an ultrahigh tunneling electroresistance (TER) exceeding 10⁸.^[^ ^132^ ^]^ More recently, a MoS_2_/α‐In_2_Se_3_/Ti FTJ device exhibited simultaneous room‐temperature negative differential resistance and a high TER over 10⁴, further validating its potential for miniaturized multifunctional electronics.^[^ ^139^ ^]^

#### 4.1.2 Group‐IV Monochalcogenides‐Based Devices

Monolayer group‐IV monochalcogenides (MX, M = Ge, Sn; X = S, Se, and Te) were theoretically predicted in 2016 to exhibit large IP spontaneous polarization.^[^ ^11^ ^]^ Monolayer MX adopts two energetically stable, inversion‐related structures, as illustrated in Figure 5f. The discovery of robust IP ferroelectricity in few‐layer SnTe by Chang et al. (2016)^[^ ^18^ ^]^ initiated intensive experimental efforts on this material family. Subsequent works demonstrated pronounced room‐temperature ferroelectricity in SnS,^[^ ^140^ ^]^ SnSe,^[^ ^92^ ^]^ GeS,^[^ ^141^ ^]^ GeSe,^[^ ^142^ ^]^ and GeTe,^[^ ^143^ ^]^ confirmed through techniques such as second harmonic generation, PFM, and scanning tunneling microscopy. These monolayers exhibit controllable domain wall motion, domain nucleation, and reversible switching, enabling excellent ferroelectric performance at the atomic scale. The outstanding IP polarization stability and scalability make MX monolayers promising for ultrathin nonvolatile memories and neuromorphic devices. For example, Wang et al. fabricated ≈28 nm thick SnSe ferroelectric films via pulsed laser deposition and developed Au/SnSe/NSTO memristors with synapse‐like structures (Figure 5g).^[^ ^144^ ^]^ The devices exhibit clear bipolar resistive switching (Figure 5h) and emulate synaptic functions through pulse modulation (Figure 5i), achieving current saturation with 800 ns pulses and a minimum energy consumption of 66 fJ.

#### 4.1.3 Bi_2_O_2_Se ‐Based Devices

Bi_2_O_2_Se is an emerging 2D ferroelectric semiconductor that has attracted attention due to its tunable structure and properties, as well as its potential in electronics, optoelectronics, and energy devices.^[^ ^145^ ^]^ It is an intrinsic n‐type quasi‐2D semiconductor with a layered body‐centered tetragonal structure (*a* = *b* = 3.887 Å, *c* = 12.164 Å), belonging to the I4/mmm space group (Figure 5j). Although the pristine Bi_2_O_2_Se structure is centrosymmetric, strain can induce piezoelectric or ferroelectric behavior. As shown in Figure 5j (right), in‐plane biaxial strain distorts the lattice, causing Se/O and Bi atoms to shift in opposite directions, resulting in a net polarization along the diagonal. Polarization appears when strain exceeds 1.7% and increases with further strain, reaching 56.1 µC cm^−^^2^ at 4.1%.^[^ ^146^ ^]^ Since the theoretical prediction of ferroelectricity in Bi_2_O_2_Se in 2017, experimental investigations have progressively followed. In 2019, Ghosh et al. fabricated free‐standing Bi_2_O_2_Se nanosheets with a thickness of 2 nm.^[^ ^21^ ^]^ Ferroelectricity was confirmed by hysteresis behavior observed through PFM and local structural distortions revealed by atomic‐resolution STEM. In 2023, Wang et al. developed a Bi_2_O_2_Se FET with an on/off ratio of 10⁴ and a 47% memory window.^[^ ^43^ ^]^ Wu et al. observed typical butterfly amplitude curves and 180° phase switching in PFM under a 700 nN tip load,^[^ ^121^ ^]^ with a switching ratio of ≈10⁶. In 2024, Khan et al. used CVD to synthesize 6 nm thick Bi_2_O_2_Se flakes and built a FET with a 47.7 µm channel length (Figure 5k),^[^ ^147^ ^]^ showing a high on/off ratio of 10⁸ and a mobility of ≈131 cm^2^ V^−1^ s^−1^. PFM patterning showed clear 180° phase contrast (Figure 5l), confirming good ferroelectric switching. In 2025, Wan et al. demonstrated a planar Bi_2_O_2_Se memristor with over 28 000 switching cycles, fast 400 µs switching, and nearly linear LTP and LTD behavior (Figure 5m),^[^ ^148^ ^]^ making it promising for energy‐efficient computing and memory applications.

#### 4.1.4 CuInP_2_S_6_‐Based Devices

CIPS is also a typical 2D ferroelectric semiconductor, where OOP polarization arises from the asymmetric vertical displacement of Cu and In ions. This relative displacement induces spontaneous polarization perpendicular to the basal plane. As a wide‐bandgap semiconductor with insulating electrical characteristics, CIPS is suitable for use as a gate dielectric or tunneling/barrier layer in ferroelectric devices. CIPS has been widely applied in ferroelectric memristors, ferroelectric diodes (FDs), FTJs, and FeFETs. For example, Ag/CIPS/Au‐based memristors (Figure 5o,p) exhibit excellent non‐volatile memory behavior, with an on/off ratio of 10^3^ and data retention exceeding 10⁴ s,^[^ ^135^ ^]^ enabling both selection and memory functions.

In a CIPS/Si heterostructure FD, Liu et al. demonstrated an on/off ratio of ≈100,^[^ ^13^ ^]^ comparable to oxide‐based devices, with resistive switching clearly observed at 1.3 V due to polarization reversal in the CIPS layer. Similar switching performance was also observed in Au/CIPS/Cr and Au/CIPS/Ni metal–ferroelectric–metal structures, with on/off ratios up to 10^3^.^[^ ^149^ ^]^ In FTJs, CIPS as a ferroelectric barrier layer, combined with asymmetric graphene/Cr electrodes, enabled tunneling resistance ratios exceeding 10⁷,^[^ ^28^ ^]^ as shown in Figure 5q. Further enhancement was achieved by inserting monolayer MoS_2_ or WSe_2_ at the CIPS/graphene interface, yielding van der Waals FTJs with TER ratios above 10^10^,^[^ ^150^ ^]^ significantly surpassing that of conventional FTJs. In FeFETs, replacing conventional ferroelectrics with CIPS improves interface quality and integration density. A MoS_2_/CIPS van der Waals FeFET showed a subthreshold swing (SS) below the Boltzmann limit across seven decades of drain current, with a minimum SS of 28 mV dec^−1^,^[^ ^151^ ^]^ indicative of negative capacitance behavior. Furthermore, when MoS_2_ is replaced by the ferroelectric semiconductor In_2_Se_3_,^[^ ^131^ ^]^ the interfacial dipole coupling between CIPS and In_2_Se_3_ leads to enhanced electrical performance. The resulting CIPS/α‐In_2_Se_3_ heterostructure device exhibits a wide memory window, an on/off current ratio exceeding 10⁶, stable data retention over 10⁴ s, and endurance over 10⁴ switching cycles.

### 4.2 Optoelectronics

2D ferroelectric semiconductors switchable polarization fields can modulate band alignment, carrier dynamics, and light–matter interactions, enabling reconfigurable photodetection, optical memory, and neuromorphic computation. Compared with conventional bulk ferroelectrics, 2D FeSCs offer tunable band gaps across the visible–infrared range, strong light–polarization coupling, and intrinsic multifunctionality, allowing them to simultaneously serve as the light absorber, ferroelectric layer, and conductive channel in a single device.

#### 4.2.1 Electro–Optical Coupling in α‐In_2_Se_3_

A prototypical example is α‐In_2_Se_3_, which exhibits robust room‐temperature ferroelectricity and a moderate bandgap (1.3–1.5 eV).^[^ ^12^ ^]^ Its coupled ferroelectric and semiconducting nature allows simultaneous optical and electrical modulation within one material. In photodetectors, α‐In_2_Se_3_ delivers a photoresponsivity as high as 2.86 × 10⁶ A W^−1^ and detection sensitivity down to ≈20 photons, owing to the synergistic effect of ferroelectric and photovoltaic responses.^[^ ^152^ ^]^ It also demonstrates long data retention (>10 years), high on/off ratio (≈2.9 × 10⁵), and endurance beyond 10⁶ cycles, suitable for nonvolatile optoelectronic memory.

**Figure 6:**
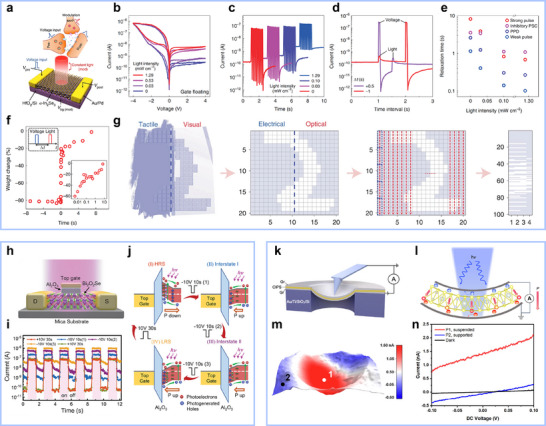
Applications of ferroelectric semiconductors in optoelectronics. a) Modulation of the third terminal of the α‐In_2_Se_3_ bipolar electric synapse by constant illumination/back‐gate voltage. b) Relationship between ferroelectric memristor switching and light intensity. c) Inhibitory PSC induced by electrical pulses (−1 V, 100 ms, 6.7 Hz) decreases under constant illumination (0–1.29 mW cm^−2^) due to shorter relaxation time and reduced accumulation. d) Timing interaction between electrical (−2 V, 50 ms) and optical (1.29 mW cm^−2^, 50 ms) pulses at −0.1 V bias, with Δ*t* = *t*_light_ − *t*_electrical_. e) Relaxation time decreases with increasing light intensity under varying electrical stimuli. f) Weight changes at different intervals between electrical and optical pulses. g) α‐In_2_Se_3_ synapse for multimodal signal processing. Reproduced with permission.^[^ ^42^ ^]^ Copyright 2022, Springer Nature. h) Schematic of the Bi_2_O_2_Se photodetector. i) FeS‐PD photoresponse under 405 nm, 1.1184 mW cm^−2^ illumination after withdrawal of different gate pulses. j) Band diagrams of FeS‐PD in HRS, intermediate states I/II, and LRS after gate pulse withdrawal, showing enhanced electron–hole separation under 405 nm light due to remanent polarization. Reproduced with permission.^[^ ^43^ ^]^ Copyright 2023, John Wiley and Sons. k) Schematic of the experimental setup for measuring the suspended CIPS. l) Structural schematic of CIPS under bending deformation. m) Short‐circuit photocurrent mapping collected by graphene electrodes on suspended and substrate‐supported regions. n) Single‐point *I*–*V* curves measured at the white and black markers in (m), corresponding to the suspended region (red) and the substrate‐supported region (blue), respectively. Reproduced with permission.^[^ ^40^ ^]^ Copyright 2024, American Chemical Society.

Liu et al.^[^ ^42^ ^]^ developed a photonic synaptic device based on α‐In_2_Se_3_ (**Figure** **6**a), achieving for the first time electro–optical dual modulation of synaptic plasticity. As shown in Figure 6b, increasing the light intensity from 0 to 1.29 mW cm^−2^ leads to a significant increase in the current levels during both set and reset processes, due to the injection of photogenerated carriers. In addition, the inhibitory postsynaptic current (PSC), triggered by electrical pulses under varying light intensities, gradually diminishes with increasing light intensity, which is attributed to shortened relaxation times and reduced accumulative effects (Figure 6c). Figure 6e further confirms a negative correlation between relaxation time and light intensity, indicating that stronger illumination can effectively accelerate the dynamic recovery of the device. The temporal interplay between optical and electrical pulses is illustrated in Figure 6d. When a light pulse precedes an electrical pulse (Figure 6d,f), optical memory can be completely erased by subsequent electrical input, highlighting the reversibility of photoinduced states. These results enabled a multimodal neuromorphic system capable of sensory fusion and temporal learning (Figure 6g). In addition, Uzhansky et al. utilized the bulk photovoltaic effect (BPVE) of α‐In_2_Se_3_ to realize a self‐powered, nonvolatile photovoltaic memory operating without external bias.^[^ ^26^ ^]^

#### 4.2.2 Polarization‐Enhanced Photodetection in MX

Group‐III/IV monochalcogenides such as InSe, SnSe, and GeSe combine ferroelectricity with excellent charge transport, making them promising for polarization‐tunable photodetection.

A pure InSe photodetector achieved a responsivity of 2300 A W^−1^ at 1 V bias and 90 mA W^−1^ even at zero bias, with a 56 ms response time and detectivity of 2.8 × 10  Jones.^[^ ^153^ ^]^ Ferroelectric gating further enhances performance: in a β‐InSe/graphene heterostructure, Li et al. (2023) reported a sixfold detectivity enhancement under negative polarization relative to the unpolarized state.^[^ ^154^ ^]^ More recently, Wang et al. (2024) introduced sliding‐induced ferroelectricity into ε‐InSe, achieving a picosecond‐scale bulk photovoltaic response in a vertical graphene/ε‐InSe/graphene heterostructure under near‐infrared illumination, further confirming the significant potential of ferroelectric polarization in boosting photodetector performance.^[^ ^155^ ^]^ Moreover, several studies have explored heterostructures composed of MX materials and conventional ferroelectrics, utilizing ferroelectric polarization to modulate their photoresponse. For instance, Weng et al. reported a CuCrP_2_S_6_/InSe heterostructure that achieved a responsivity of 1839 A W^−1^ and a detectivity of 1.9 × 10^−2^ Jones at a wavelength of 300 nm, with a responsivity contrast of up to 20.7 times between different polarization states.^[^ ^156^ ^]^

#### 4.2.3 Polarization‐Enhanced Photodetection in Bi_2_O_2_Se

Bi_2_O_2_Se is another 2D ferroelectric semiconductor with a small indirect bandgap (≈0.8 eV) and high electron mobility, suitable for broadband optoelectronics. However, its narrow gap often causes large dark currents, which can be mitigated via heterojunction or interface engineering. Tao et al. combined Bi_2_O_2_Se with MoSe_2_ to form a type‐II junction that reduced dark current through interlayer charge separation;^[^ ^157^ ^]^ Huang et al. fabricated a Bi_2_O_2_Se/Si photodetector with low dark current (22.3 nA cm^−2^), high on/off ratio (8 × 10⁶), and responsivity of 23 A W^−1^;^[^ ^158^ ^]^ and asymmetric electrodes have been shown to further suppress leakage.^[^ ^159^ ^]^ Additionally, Bi_2_O_2_Se's ferroelectricity can be used to tune device performance. Wang et al.^[^ ^43^ ^]^ demonstrated polarization‐controlled photodetection in Bi_2_O_2_Se, where ferroelectric switching modulates both photocurrent and dark current. Figure 6h presents a schematic of the photodetector (FeS‐PD) based on Bi_2_O_2_Se, which can switch between different optical response states using gate pulses. The influence of polarization on the photodetection performance was studied by switching between different optical response states with gate pulses. Figure 6i illustrates the time‐dependent optical response at various polarization levels. The photodetector operates by switching ferroelectric polarization, creating an internal electric field that enhances residual polarization. Applying gate pulses bends the energy bands, altering channel resistance, and adjusting both photocurrent and dark current. Positive gate pulses set the channel to HRS, while negative pulses decrease resistance, increasing photocurrent and dark current. Figure 6e shows the band diagram of the FeS‐PD under residual polarization. Gate pulse adjustment allows precise control of photonic responses by switching between bistable resistance states (HRS, State I, State II, LRS). This photodetector offers highly tunable performance, a simplified structure, and potential for integrating high‐performance optoelectronic devices.

#### 4.2.4 Bulk Photovoltaic Effect and Flexoelectric Enhancement

The BPVE—the generation of a steady photocurrent in a non‐centrosymmetric crystal without external bias—is a hallmark of ferroelectrics, originating from shift current and asymmetrical excitation processes. Initially observed in ferroelectric oxides, BPVE has since been extensively explored in emerging materials such as Weyl semimetals.^[^ ^160^ ^]^ vdW nanomaterials,^[^ ^122^ ^]^ oxide superlattices,^[^ ^161^ ^]^ halide perovskites,^[^ ^162^ ^]^ organic compounds,^[^ ^163^ ^]^ and bulk Rashba semiconductors.^[^ ^164^ ^]^ In 2D FeSCs, BPVE has been experimentally confirmed and can be amplified through strain or flexoelectric coupling. CIPS serves as a representative case. Li et al.^[^ ^25^ ^]^ used CIPS as a photoferroelectric layer sandwiched by graphene electrodes, observing a two‐orders‐of‐magnitude photocurrent enhancement compared with bulk ferroelectrics. Building on this, Yu et al. enhanced the BPVE in CIPS via the flexoelectric effect and demonstrated its mechanical tunability.^[^ ^40^ ^]^ By transferring CIPS onto a perforated substrate and applying local strain using a PFM tip (Figure 6k), they broke the crystal symmetry (Figure 6l), driving Cu⁺ ions toward polarized lattice sites and inducing uniform polarization. The short‐circuit current mapping under 405 nm laser illumination (Figure 6m) showed a peak current of 1.43 nA at the suspended region (Point 1), while the supported region (Point 2) exhibited only –65.9 pA (Figure 6n), confirming that flexoelectric strain effectively boosts the BPVE in CIPS. Similarly, Dong et al.^[^ ^122^ ^]^ reported strain‐enhanced photocurrents in non‐centrosymmetric MoS_2_, and Wang et al.^[^ ^165^ ^]^ demonstrated polarization‐switchable BPVE in α‐In_2_Se_3_. Peng et al. achieved highly sensitive polarization‐resolved photodetection in a trilayer hybrid perovskite ferroelectric, (allyammonium)_2_(ethylammonium)_2_Pb_3_Br_10_, exhibiting a near‐bandgap open‐circuit voltage of ≈2.5 V, an on/off ratio of 10⁴, and a polarization ratio up to 15.^[^ ^166^ ^]^

### 4.3 Spintronics and Valleytronics

The spin and valley degrees of freedom introduce new dimensions for tuning device performance. In 2D materials, where reduced symmetry and strong spin–orbit coupling (SOC) prevail, these internal degrees of freedom can be sensitively tuned by structural or electrical perturbations. Ferroelectric semiconductors, with their switchable spontaneous polarization and intrinsic coupling among charge, lattice, orbital, and spin, naturally provide a powerful platform for manipulating spin and valley states via electric fields. Such coupling not only enables efficient spin and valley control without magnetic fields but also promotes the integration of multiple information modalities—charge, spin, and valley—within a single architecture.

#### 4.3.1 Ferroelectric Control of Spin and Magnetism

In spintronics, ferroelectric polarization–induced Rashba spin splitting introduces spin‐dependent band dispersion, enabling electrical control over spin degrees of freedom. This is crucial for devices like spin field‐effect transistors. Compared with conventional spintronic devices relying on magnetic field manipulation, electric control offers low power consumption and better integration. Moreover, the coupling between ferroelectricity and SOC allows reversible spin polarization switching, supporting non‐volatile spin storage and logic. Some 2D van der Waals ferroelectrics exhibit large Rashba effects, with Rashba parameters surpassing those from conventional surface or interface effects. For example, Bruyer et al.^[^ ^167^ ^]^ found that trilayer MoS_2_, with d^2^ metal ions, retains switchable Rashba spin textures even at the monolayer limit.

Building on this concept, ferroelectric Rashba semiconductors have emerged as a key materials class that combines semiconducting transport, strong SOC, and ferroelectric nonvolatility. In Fe/GeTe heterostructures, Varotto et al.^[^ ^45^ ^]^ reported that spin‐to‐charge conversion efficiency can be reversibly tuned by flipping ferroelectric polarization, thereby demonstrating nonvolatile spin manipulation purely through electric means. These findings highlight a unifying principle across diverse systems: the electrostatic field associated with ferroelectric polarization directly modifies the local potential gradient and, consequently, the spin texture and spin current response—an effect central to scalable spintronic logic and memory design.

#### 4.3.2 Multiferroic Coupling and 2D Magnetic–Ferroelectric Integration

**Figure 7:**
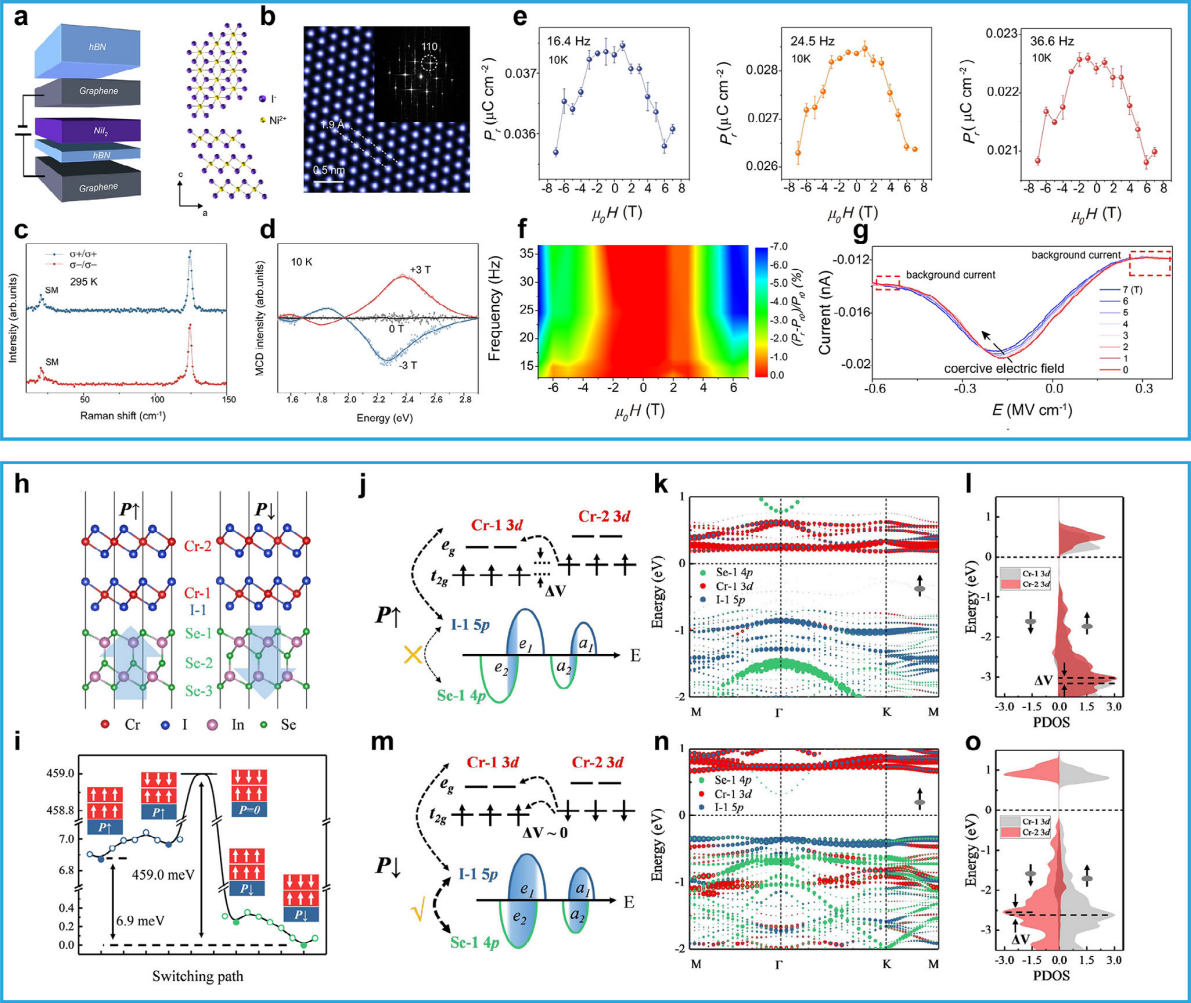
a) Schematic of trilayer NiI_2_ sandwiched between graphene and hBN, showing both IP and OOP atomic lattice structures. Ni^2^⁺ ions are coordinated by I^−^ octahedra, and the trilayer NiI_2_ stacks in a staggered fashion along the *c‐*axis. b) Atomic‐resolution ADF‐STEM image displaying the hexagonal pattern characteristic of rhombohedral stacking in few‐layer NiI_2_ crystals; inset shows the corresponding FFT image. c) Circular polarization‐resolved Raman spectra of the NiI_2_ device under 532 nm laser excitation, highlighting the interlayer shear mode (SM). d) MCD spectra of trilayer NiI_2_ measured under magnetic fields of +3 T, 0 T, and −3 T. e) Remanent polarization (*P*_r_) as a function of OOP magnetic field at different frequencies. f) Phase diagram of the magnetic control ratio, (*P*_r_ *− P*_r0_)/*P*_r0_, as a function of frequency and magnetic field, where *P*_r_ and *P*_r0_ represent the remanent polarization with and without a magnetic field, respectively. g) *I*–*E* curves measured under different magnetic fields.^[^ ^168^ ^]^ Copyright 2024, The Authors, published by Springer Nature. h) Side view of bi‐CrI_3_/In_2_Se_3_. i) Switching path between the FM‐*P*↑ and AFM‐*P*↓ states in the high‐temperature phase of bi‐CrI_3_/In_2_Se_3_. Blue and green circles represent different magnetic states in the *P*↑ and *P*↓ configurations, respectively. When *P* = 0, the system is non‐ferroelectric. j,m) Schematic diagrams of orbital coupling between the interfacial I‐1 and Se‐1 p orbitals, and the virtual hopping path between the empty Cr‐1 *e*_g_ and occupied Cr‐2 *t*_2g_ orbitals. k,n) Orbital‐projected band structures (spin‐up channel). l,o) Projected density of states (PDOS). Reproduced with permission.^[^ ^23^ ^]^ Copyright 2024, American Physical Society.

Beyond Rashba‐type effects, intrinsic 2D multiferroics offer direct coupling between electric and magnetic order parameters, enabling cross‐control of magnetism and ferroelectricity. Although achieving room‐temperature stability remains challenging, recent experimental breakthroughs have verified genuine 2D multiferroicity. Wu et al.^[^ ^168^ ^]^ identified the coexistence of ferroelectricity and antiferromagnetism in layered NiI_2_, establishing it as a single‐phase van der Waals multiferroic through optical, electrical, and magnetic characterizations. In their study, trilayer NiI_2_ devices encapsulated with graphene and hBN (**Figure** **7**a) showed a rhombohedral stacking structure with triangular Ni–I lattices (Figure 7b). ADF‐STEM imaging confirmed the stacking order and an interlayer spacing of ≈1.9 Å (Figure 7c), while circularly polarized Raman spectra identified the A_1_g and interlayer shear modes characteristic of trilayer geometry (Figure 7d). Magnetic circular dichroism (MCD) and reflective MCD spectra revealed field‐dependent magnetic signals and bimeron‐like topological domains (Figure 7e), and frequency‐dependent P–E and I–E loops confirmed the coexistence of ferroelectric and antiferromagnetic order. The magnetoelectric coefficient reached ≈7% under 7 T at 24.5 Hz (Figure 7f), signifying a prototypical type‐II multiferroic coupling. The systematic modulation of ferroelectric switching by external magnetic fields provides compelling evidence of the dynamic interplay between spin and polarization in van der Waals materials.

A complementary strategy to engineer spin–electric coupling is the assembly of ferroelectric/ferromagnetic van der Waals heterostructures, where interfacial polarization fields mediate magnetism across atomic layers. Yang et al.^[^ ^23^ ^]^ realized such a system by integrating bilayer CrI_3_ with monolayer In_2_Se_3_ (Figure 7h). In this heterostructure, the polarization direction of In_2_Se_3_ controls the interlayer magnetic alignment of CrI_3_: the upward polarization (P↑) and downward polarization (P↓) states correspond to ferromagnetic and antiferromagnetic configurations, respectively, separated by an energy barrier of 459 meV per Cr atom (Figure 7i). Unlike charge‐transfer‐based modulation, the interfacial coupling here is electrostatic and orbital in nature, preserving the semiconducting characteristics. PDOS analysis (Figure 7j,k) shows that the P↑ induces a potential difference (Δ*V*) that stabilizes the ferromagnetic state (Figure 7l), whereas the P↓ enhances orbital hybridization (Figure 7m–o), reducing Δ*V* and favoring antiferromagnetism. This bidirectional electrical control of magnetism exemplifies a key physical insight across various systems: ferroelectric polarization acts as an internal gate that reshapes the magnetic exchange interactions through spin‐dependent orbital overlap, offering a unified route to electrically tunable spin order.

#### 4.3.3 Ferroelectric Modulation of Valley Degrees of Freedom

In valleytronics, ferroelectric polarization breaks the degeneracy between *K* and *K*′ valleys, leading to valley polarization and enabling electrical and optical control over the valley degree of freedom. Moreover, polarization‐induced Berry curvature reconstruction can give rise to the valley Hall effect, generating transverse valley currents in the absence of an external magnetic field—a promising mechanism for low‐power information processing. Initial studies focused on 2D hexagonal lattice systems.^[^ ^169^ ^]^ As research has progressed, intrinsic coupling between ferroelectric polarization and spontaneous valley polarization has been observed in group‐IV monochalcogenides, such as SnS and GeSe. For instance, GeSe^[^ ^170^ ^]^ exhibits two vertical polarization orientations, each corresponding to opposite valley polarization states. Owing to the synergy between ferroelectricity and valley polarity, the direction of valley polarization can be reversed through ferroelectric switching.

Beyond intrinsic coupling, heterostructure engineering, interlayer sliding, and ionic doping offer additional degrees of control. For example, in the MnPS_3_/CIPS heterostructure,^[^ ^171^ ^]^ ferroelectric polarization induced by proximity effects modulates the direction of valley polarization and the optical bandgap, enabling multidimensional tuning of electronic degrees of freedom.

## 5 Challenges and Future Perspectives

**Figure 8:**
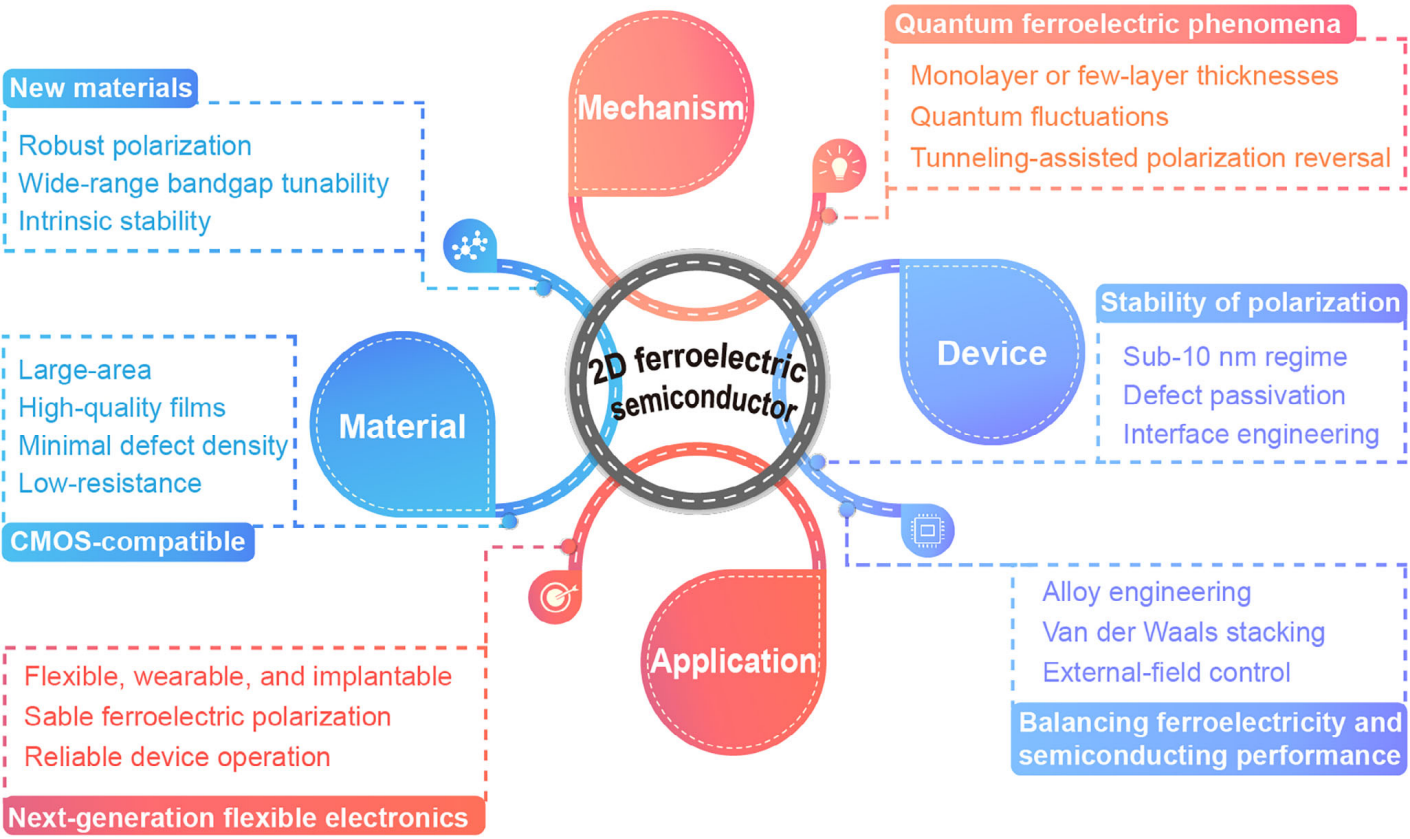
Challenges of 2D ferroelectric semiconductors.

The continued advancement of 2D ferroelectric semiconductors for next‐generation nanoelectronic and optoelectronic technologies hinges on overcoming several critical challenges, which span from fundamental scientific questions to practical engineering issues, as illustrated in **Figure** **8**.

### 5.1 Challenges

#### 5.1.1 Exploring Quantum Ferroelectric Phenomena at the Ultrathin Limit

When scaled to monolayer or few‐layer thicknesses, 2D ferroelectric semiconductors may exhibit quantum phenomena that fundamentally alter their polarization behavior. Quantum fluctuations, tunneling‐assisted polarization reversal, and proximity to quantum critical points could give rise to entirely new physics beyond classical ferroelectricity. Unraveling these effects and understanding their implications for applications in spintronics, quantum information devices, and correlated electron systems represent exciting frontiers for basic research.

#### 5.1.2 Discovery and Rapid Screening of New Materials

The library of experimentally realized 2D ferroelectric semiconductors remains relatively narrow, largely limited to a few families such as IV–VI group compounds, Janus structures, and layered oxides like Bi_2_O_2_Se. Expanding this material space is crucial for diversifying functional properties and enabling new applications. High‐throughput computational screening, accelerated by machine learning and data‐driven approaches, holds great promise for discovering novel 2D materials that combine robust polarization, wide‐range bandgap tunability, and intrinsic stability under operational conditions.

#### 5.1.3 Compatibility with Mainstream Semiconductor Technologies

For 2D ferroelectric semiconductors to make a meaningful impact in practical devices, their integration with existing CMOS technologies must be addressed. This involves not only the scalable growth of large‐area, high‐quality films with uniform properties and minimal defect density, but also the realization of low‐resistance, stable interfaces with metal contacts and insulating layers. Furthermore, the ability to transfer or directly synthesize these materials on technologically relevant substrates, while preserving their ferroelectric and semiconducting functionalities, is essential for seamless incorporation into current fabrication processes.

#### 5.1.4 Stability of Polarization over Time and with Scaling

As device dimensions are pushed into the sub‐10 nm regime, the ferroelectric polarization in 2D semiconductors becomes increasingly vulnerable to thermal fluctuations, depolarization fields, and the presence of defects. These factors can lead to significant degradation of remanent polarization and switching reliability over time. Ensuring robust, switchable polarization at room temperature in the ultrathin limit, while maintaining long‐term retention and fatigue endurance, remains a central challenge. This calls for innovative strategies in material design, defect passivation, and interface engineering to suppress depolarization and stabilize ferroelectric order at atomic thicknesses.

#### 5.1.5 Balancing Ferroelectricity and Semiconducting Performance

Achieving a harmonious integration of strong ferroelectric properties with desirable semiconducting characteristics—such as appropriate band gaps, high carrier mobility, and low trap density—is inherently challenging. Strong spontaneous polarization often induces significant lattice distortions, which in turn can reduce carrier mobility and degrade charge transport. The development of 2D ferroelectric semiconductors thus requires careful tuning of composition, strain, and heterostructure configurations to reconcile these competing requirements. Alloy engineering, van der Waals stacking, and external‐field control represent promising approaches to achieve this delicate balance.

#### 5.1.6 Potential in Next‐Generation Flexible and Wearable Electronics

The atomic thinness and mechanical flexibility of 2D ferroelectric semiconductors position them as promising candidates for flexible, wearable, and implantable electronic systems. However, maintaining stable ferroelectric polarization and reliable device operation under repeated bending, stretching, and other mechanical deformations remains a significant challenge. Progress in scalable synthesis techniques, as well as the development of robust device architectures capable of withstanding mechanical stress, will be key to realizing their full potential in these emerging applications.

### 5.2 Future Directions

Looking ahead, several emerging research avenues are expected to further expand the scientific and technological potential of 2D ferroelectric semiconductors:

#### 5.2.1 Strain Engineering for Polarization and Phase Control

The extreme mechanical flexibility of 2D materials provides a unique platform for strain engineering, enabling the precise modulation of polarization orientation, magnitude, and switching barrier. Dynamically tunable strain fields—achieved via substrate coupling, local gating, or piezoelectric actuators—may allow reversible control of ferroelectric domains, phase boundaries, and even emergent ferroelastic states, offering a pathway toward adaptive and reconfigurable devices.

#### 5.2.2 Twist‐Angle and Moiré Engineering

Twist‐angle control in van der Waals bilayers offers another promising route to realize exotic ferroelectric phenomena, such as moiré‐induced polarization, fractional domain‐wall charges, and electrically switchable dipole textures. Systematic exploration of moiré ferroelectricity, including the interplay between twist angle, stacking configuration, and interlayer coupling, will be crucial for developing next‐generation twistronic ferroelectric devices with programmable polarization landscapes.

#### 5.2.3 Integration with Quantum and Correlated Materials

Coupling 2D ferroelectric semiconductors with quantum materials—such as topological insulators, superconductors, and magnetic van der Waals layers—could lead to novel quantum functionalities arising from ferroelectric control of topological phases, spin textures, or superconducting pairing. Such hybrid architectures are expected to open exciting opportunities in nonvolatile quantum logic, topological memory, and low‐dissipation information processing.

#### 5.2.4 Artificial Heterostructures and Neuromorphic Architectures

Designing artificial van der Waals heterostructures that combine ferroelectric, semiconducting, and magnetic components may enable multifunctional devices capable of performing sensing, memory, and computation simultaneously. Beyond conventional transistors, integrating 2D ferroelectric semiconductors into neuromorphic architectures could leverage their analog switching and nonvolatile polarization states for brain‐inspired computing.

#### 5.2.5 Toward Sustainable and Scalable Integration

Finally, developing environmentally friendly synthesis routes, wafer‐scale integration strategies, and defect‐tolerant architectures will be essential for bridging the gap between laboratory demonstrations and real‐world applications. Future efforts should focus on the co‐optimization of material quality, interface design, and device performance to establish 2D ferroelectric semiconductors as key enablers in next‐generation low‐power and multifunctional nanoelectronics.

## 6 Conclusion

2D ferroelectric semiconductors have emerged as a frontier in ferroelectric materials research, offering a compelling combination of atomic‐scale thickness, spontaneous polarization, excellent semiconducting properties, and tunable band gaps. These unique features are driving a new wave of innovation in micro‐ and nanoelectronics as well as optoelectronics. This review has provided a systematic overview of the evolution of ferroelectricity, with a focus on the origin of intrinsic ferroelectricity in 2D materials, their distinctive properties compared with conventional 3D ferroelectrics, and recent advances in both intrinsic and extrinsically engineered 2D ferroelectric semiconductors.

In terms of device applications, 2D ferroelectric semiconductors exhibit significant potential in a broad range of technologies, including FeFETs, ferroelectric tunnel junctions, neuromorphic computing devices, piezotronic systems, ferroelectric photodetectors, bulk photovoltaic effect devices, piezophototronic structures, as well as spintronic and valleytronic applications. These emerging applications offer promising pathways toward the development of next‐generation high‐density, low‐power, nonvolatile memory and logic devices, while also providing a new material foundation for flexible electronics, wearable technologies, and hardware for neural network computing.

Despite the remarkable progress, the development of 2D ferroelectric semiconductors continues to face critical challenges. Key issues include ensuring polarization stability at reduced dimensions and over extended operating lifetimes, achieving integration compatibility with existing silicon‐based semiconductor processes, balancing strong ferroelectric properties with high carrier mobility and tunable electronic structures, and accelerating the discovery of new materials through high‐throughput screening and machine learning approaches. Future research must not only advance material design, modulation, and scalable fabrication techniques, but also explore quantum ferroelectric phenomena at the ultimate thickness limit and their potential in flexible and wearable electronic systems.

In summary, as a new class of materials combining intrinsic ferroelectricity with outstanding semiconducting performance, 2D ferroelectric semiconductors are steadily advancing the frontiers of nanoelectronics, optoelectronics, and spintronics. Their application in next‐generation information technologies warrants continued and intensive investigation.

## Conflict of Interest

The authors declare no conflict of interest.

## Author Contributions

Conceptualization was done by M.C. and J.Z. Original Draft was written by M.C. M.C. and D.G. prepared the figures. M.C., X.Z., J.L., Y.W., and A.Y. were responsible for analysis and synthesis. Funding Acquisition was done by J.Z. and D.G. Supervision was done by J.Z. and D.G. All authors revised and approved the final version of the manuscript.
